# Metal to non-metal sites of metallic sulfides switching products from CO to CH_4_ for photocatalytic CO_2_ reduction

**DOI:** 10.1038/s41467-023-41943-x

**Published:** 2023-10-04

**Authors:** Yao Chai, Yuehua Kong, Min Lin, Wei Lin, Jinni Shen, Jinlin Long, Rusheng Yuan, Wenxin Dai, Xuxu Wang, Zizhong Zhang

**Affiliations:** 1 State Key Lab of Photocatalysis on Energy and Environment, College of Chemistry, Fuzhou University, Fuzhou, P. R. China; 2 College of Chemistry, Fuzhou University, Fuzhou, P. R. China; 3 Qingyuan Innovation Laboratory, Quanzhou, P. R. China

**Keywords:** Photocatalysis, Energy, Structural properties

## Abstract

The active center for the adsorption and activation of carbon dioxide plays a vital role in the conversion and product selectivity of photocatalytic CO_2_ reduction. Here, we find multiple metal sulfides CuInSnS_4_ octahedral nanocrystal with exposed (1 1 1) plane for the selectively photocatalytic CO_2_ reduction to methane. Still, the product is switched to carbon monoxide on the corresponding individual metal sulfides In_2_S_3_, SnS_2_, and Cu_2_S. Unlike the common metal or defects as active sites, the non-metal sulfur atom in CuInSnS_4_ is revealed to be the adsorption center for responding to the selectivity of CH_4_ products. The carbon atom of CO_2_ adsorbed on the electron-poor sulfur atom of CuInSnS_4_ is favorable for stabilizing the intermediates and thus promotes the conversion of CO_2_ to CH_4_. Both the activity and selectivity of CH_4_ products over the pristine CuInSnS_4_ nanocrystal can be further improved by the modification of with various co-catalysts to enhance the separation of the photogenerated charge carrier. This work provides a non-metal active site to determine the conversion and selectivity of photocatalytic CO_2_ reduction.

## Abstract (web summary)

The product selectivity of photocatalytic carbon dioxide reduction from carbon monoxide to methane is determined by the active center from metal to sulfur site in metal sulfides. Non-metal sulfur in CuInSnS4 octahedral nanocrystal acts as carbon dioxide activation center for switching selectivity to methane.

## Introduction

The solar-energy-driven photocatalytic conversion of CO_2_ with H_2_O into hydrocarbon fuels is a significant solution for simultaneously addressing global energy demands and climate change issues^1–4^. Various products of CO_2_ reduction from photocatalytic multi-electron processes, including CO (two electrons), HCOOH (two electrons), HCHO (four electrons), CH_3_OH (six electrons), and CH_4_ (eight electrons), have been produced by a great variety of photocatalysts^5–8^. Achieving both high selectivity and high conversion for photocatalytic CO_2_ reduction is highly desirable in the field of photocatalysis research. However, efficient photoreduction of CO_2_ is very challenging, both in terms of chemical thermodynamics and kinetics, due to the highly stable structure of CO_2_ and the involvement of multiple proton-coupled electron transfer^9–11^. Additionally, the regulation of product selectivity in photocatalytic CO_2_ conversion remains an unknown challenge.

It has been well understood for the photocatalytic process that the identification of the active centers of catalysts for the adsorption and activation of CO_2_ is prerequisite for efficient CO_2_ conversion and product selectivity. Constructing an active center of catalysts for the adsorption and activation of CO_2_ is an efficient solution to improve CO_2_ conversion efficiency and product selectivity^12–14^. Various metal-free photocatalysts were reported for CO_2_ reduction^15–19^, typically such as covalent organic frameworks, graphitic carbon nitride, elemental phosphorus, boron nitride, and silicon carbide^20–24^. These metal-free photocatalysts have non-metallic sites as the adsorption and activation sites of CO_2_ molecules and thus photocatalytical CO_2_ reduction^20,25–28^. However, for metal oxide or sulfide photocatalysts, many studies suggest that metal components or defects on photocatalysts play a crucial role as primary sites in the adsorption and activation of CO_2_ and thus affect product selectivity^29^. Zhou et al. reported that the S vacancy or Cd vacancy CdS with single Au atom deposition for CO_2_ adsorption is different^30^. CO_2_ prefers to physically adsorb on single Au atoms of Au/CdS_1−x_ and photoreduction into CO, while CO_2_ is more likely to chemically bond on the Cd vacancies of Au/Cd_1−x_S, resulting in a remarkable CO and CH_4_ generation rate on Au/Cd_1−x_S. He et al. synthesized a ZnIn_2_S_4_ nanosheet photocatalyst with abundant Zn vacancies^31^, where CO_2_ can be efficiently adsorbed on Zn vacancies to form CO_2_^−^ species and highly selective photoreduction into CO. Yu et al. designed a Cu_3_SnS_4_ photocatalyst with S vacancies to increase ratios of Cu (I/II) for CO_2_ photoreduction^32^. The formed Cu (I) acts as adsorption sites for CO_2_, conducive to further hydrogenation of CO intermediate into CH_4_. Xie et al. showed that the defect-state CuIn_5_S_8_ ultrathin nanosheets have low-coordination Cu and In sites for CO_2_ adsorption to form highly stable Cu-C-O-In intermediates, which tend to obtain 100% CH_4_ selectivity^33^. Xu et al. designed a Co-Ni-P NH/BP catalyst with bimetallic sites to form a highly stable Co-O-C-Ni intermediate for the selective photoreduction of CO_2_ to CH_4_^34^. However, the complex structures of defects on photocatalyst make it only a plausible correlation between defect structures and product selectivity. Some research shows that the adsorbed interaction between CO_2_ and metal sites is relatively weak since the formed metal-C bonds are weaker than the highly stable C=O bonds in CO_2_. This leads to the easy cleaving of metal-C bonds during the reaction process, hindering the deep reduction of CO_2_ into hydrocarbons^30^. Obviously, the non-metal sites on metal sulfide photocatalysts are very rarely considered the primary active center for the adsorption and activation of CO_2_.

Here, we have successfully prepared multiple metal sulfides, including CuInSnS_4_ octahedral nanocrystal and corresponding individual metal sulfides In_2_S_3_, SnS_2_, and Cu_2_S, through a simple one-step hydrothermal method. The CuInSnS_4_ nanocrystal is thermodynamically favorable to activate CO_2_ and leads to a switch of main products from CO to CH_4_ with a yield of 6.53 μL h^−1^ for the visible-light-driven CO_2_ reduction with H_2_O vapor without the assistance of any noble metal cocatalysts. In contrast, individual metal sulfides can only produce CO. We reveal that different adsorption configurations of CO_2_ on metal sulfides lead to different products in CO_2_ photoreduction. The non-metal sulfur atom in the prepared multiple metal sulfides CuInSnS_4_ octahedron nanocrystal is thermodynamically favorable to activate CO_2_ and leads to a switch of main products to CH_4_, as compared with the common individual metal sulfides In_2_S_3_, SnS_2_ and Cu_2_S with metal center as active sites to form CO products. CO_2_ is revealed to be adsorbed on the S atom center of CuInSnS_4_ to form an S-C-O-In structural unit, which is more conducive to protonation and leads to the efficient photocatalytic yield of CH_4_. Thus, we provide an insight into the role of non-metal center of photocatalyst in determining the conversion and selectivity of photocatalytic CO_2_ reduction. Although the pristine CuInSnS_4_ only exhibits a yield of CH_4_ evolution of 6.53 μL h^−1^ (corresponding to 5.83 μmol h^−1^ g^−1^), the activity and selectivity of CH_4_ evolution on CuInSnS_4_ can be significantly improved by modifying with cocatalysts such as Pt, CoO, NiO, and Co(OH)_2_. We believe that this knowledge can contribute to the development of more efficient and selective photocatalysts for CO_2_ reduction in the future.

## Results

### Characterization of CuInSnS_4_ octahedral nanocrystal

**Fig. 1:**
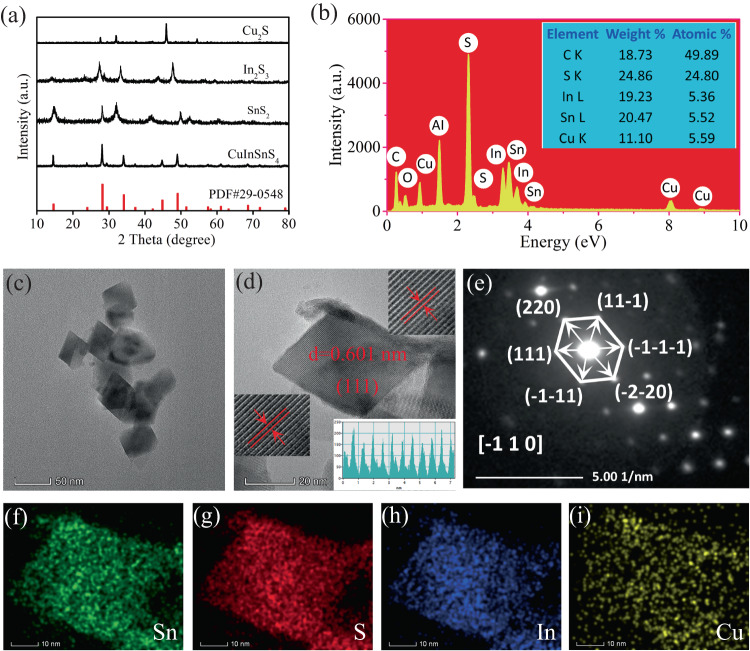
The characterization of crystal phase, composition, and morphology. **a** XRD patterns of several metal sulfides, **b** EDS spectra of CuInSnS_4_ photocatalyst, **c** a TEM image of CuInSnS_4_ sample, **d** an HRTEM image, and **e** an SAED pattern of the CuInSnS_4_ sample, as well as **f**–**i** EDS elemental mapping images.

The as-prepared CuInSnS_4_ nanocrystals belong to the cubic spinel structure (JCPDS No. 29-0548), as revealed by the X-ray diffraction pattern (Fig. 1a), which were prepared through a simple one-step hydrothermal reaction. The hydrothermal temperature (160 °C, 180 °C, and 200 °C) did not have any evident impact on the crystalline and purity of CuInSnS_4_ (Supplementary Fig. 1). Under similar hydrothermal processes, In_2_S_3_ with a tetragonal crystal phase structure (JCPDS No. 25-0390), SnS_2_ with a hexagonal crystal phase (JCPDS No. 23-0677), and Cu_2_S with a cubic crystal phase were also prepared (JCPDS No. 02-1284) (Supplementary Fig. 2). The cubic spinel CuInSnS_4_ crystal structure shows that either In or Sn atom is coordinated with six S atoms to form an octahedron structure, while Cu atom is formed by a [CuS_4_] tetrahedral structural unit (Supplementary Fig. 3). For comparison, in In_2_S_3_ crystals, the In atom is present in [InS_4_] tetrahedron and [InS_6_] octahedron coordination (Supplementary Fig. 4). Cu and Sn atoms exist as [CuS_4_] tetrahedron and [SnS_6_] octahedron coordination in Cu_2_S and SnS_2_ crystal structure, respectively (Supplementary Figs. 5, 6). The coordination structure of each metal in CuInSnS_4_ coincides with the individual Cu_2_S, In_2_S_3_, and SnS_2_. The composition of CuInSnS_4_ is analyzed by energy-dispersive spectroscopy, as shown in Fig. 1b. EDS offers that the Cu, Sn, In, and S atomic ratio is ~1:1:1:4, very close to the stoichiometric value of CuInSnS_4_ compounds, indicating the high purity of CuInSnS_4_ nanocrystal. Meantime, the ICP-MS test results of the metal element content of the nano-single crystal CuInSnS_4_ sample are presented in Table S1. The atomic ratio of Cu:In:Sn is 1.06:1.00:1.00, which closely matches the theoretical value of CuInSnS_4_. Supplementary Fig. 7 shows the SEM images of In_2_S_3_, Cu_2_S, and SnS_2_ samples. In_2_S_3_ exhibits a morphology of microspheres self-assembled from nanosheets. Cu_2_S has the basic shape of nanoparticles, while SnS_2_ displays the morphology of ultrathin nanosheets. Both scanning electron microscopy and transmission electron microscopy images of nanoscale microstructure confirm that the prepared CuInSnS_4_ displays an octahedral nanocrystal structure with a size of about 30 nm (Supplementary Fig. 8 and Fig. 1c). The high-resolution TEM image (Fig. 1d) shows the exposed facets of the octahedron with a lattice spacing of 0.601 nm, which is assigned to (1 1 1) facets of CuInSnS_4_. Selected area electron diffraction further verifies that the CuInSnS_4_ sample not only exposes the (1 1 1) crystal plane but also has a single crystal structure (Fig. 1e). All the results confirm the successful preparation of CuInSnS_4_ nanocrystal with high-quality exposed (1 1 1) crystal faces. The specific surface area of the CuInSnS_4_ sample is about 24.1 m^2^ g^−1^, while that of the prepared In_2_S_3_, SnS_2_, and Cu_2_S are 26.8 m^2^ g^−1^, 37.6 m^2^ g^−1^, and 3.6 m^2^ g^−1^, respectively (Supplementary Fig. 9). As displayed in Fig. 1f–i, the uniform distribution of Sn, S, In, and Cu elements in CuInSnS_4_ octahedral nanoparticles indicates that the catalyst is of high purity.

**Fig. 2:**
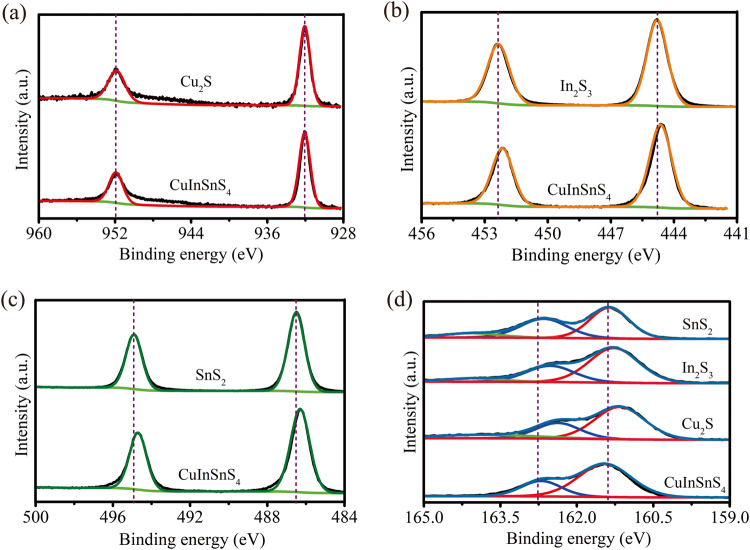
Characterization of electronic structure. High-resolution XPS spectra of metal sulfides: **a** Cu*2p*, **b** In*3d*, **c** Sn*3d*, and **d** S*2p*.

X-ray photoelectron spectroscopy was used to compare the electronic states of the obtained sample. The Cu*2p*_3/2_ and Cu*2p*_1/2_ binding energies of CuInSnS_4_ sample are 932.07 eV and 951.90 eV, respectively (Fig. 2a). This demonstrates that the valence state of Cu is +1 in the CuInSnS_4_ sample^35,36^, which is also confirmed by the Cu LMM spectra (Supplementary Fig. 10). Notably, the Cu*2p*-binding energies of the CuInSnS_4_ sample is identical to that of Cu_2_S. The binding energies of In*3d*_5/2_ and In*3d*_3/2_ in the CuInSnS_4_ sample are 444.63 eV and 452.18 eV, respectively. These values indicate that the valence state of In in the CuInSnS_4_ sample is +3. Compared with In_2_S_3_, the In*3d* binding energy of CuInSnS_4_ uniformly shifts toward the lower binding energy, as shown in Fig. 2b. This is attributed to the difference in the In coordinated environment between CuInSnS_4_ and In_2_S_3_ because the partial In atom in In_2_S_3_ exists in the state of [InS_4_] tetrahedron^37^. In the CuInSnS_4_ sample, the Sn*3d*_5/2_ and Sn*3d*_3/2_ doublets are centered respectively at 486.30 eV and 494.70 eV, assigning to Sn^4+^ valence state. Notably, the binding energy of Sn in CuInSnS_4_ is slightly lower than that in the parent SnS_2_ (Fig. 2c). The possible reason is that the Sn atoms are in different crystal structures^38^. Furthermore, the binding energies of S*2p*_3/2_ and S*2p*_1/2_ in the CuInSnS_4_ sample are measured to be 161.45 eV and 162.70 eV, respectively, which corresponds to the S^2−^ valence state. In the S*2p* XPS spectra, the binding energies of S atoms increase in the order of Cu_2_S < In_2_S_3_ < SnS_2_ < CuInSnS_4_, as shown in Fig. 2d. S atoms in CuInSnS_4_ have the highest binding energy. This is interpreted by the fact that the average bond length (0.253 nm) between sulfur and metal atoms in CuInSnS_4_ is slightly larger than in monometallic sulfide^4,37,39^. Moreover, the S atom in CuInSnS_4_ has a higher binding energy than that of monometallic sulfides, indicating an electron-deficient state of the S atoms in CuInSnS_4_ compared to monometallic sulfides. This electron-deficient state of the S atom in CuInSnS_4_ can serve as the reaction site for CO_2_ adsorption and activation.

### Photocatalytic conversion of CO_2_ and H_2_O vapor

**Fig. 3:**
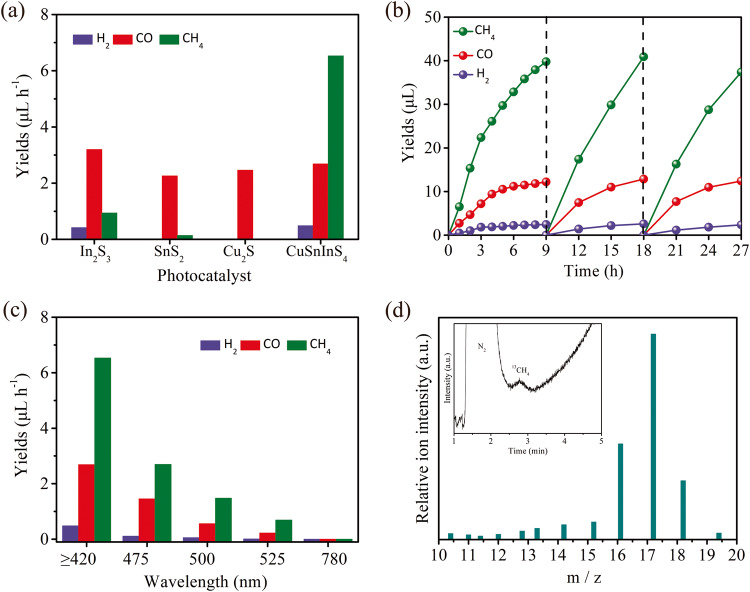
Photocatalytic conversion performance of CO_2_ and H_2_O. **a** The production rate of CH_4_, CO, and H_2_ in various photocatalysts under visible-light conditions. **b** Photocatalytic CO_2_ reduction stability test of CuInSnS_4_ sample. **c** Photocatalytic performance of CuInSnS_4_ sample under monochromatic light irradiation. **d** GC-MS spectra of ^13^CH_4_ generated from ^13^CO_2_.

The photocatalytic CO_2_ reduction performance of the samples was evaluated in a customized sealed quartz glass vessel, in a gas-solid reaction system, with a small amount of water vapor in a CO_2_ atmosphere, under the irradiation of a 300 W Xe lamp with a 420 nm cutoff wavelength filter (Supplementary Fig. 11). It is important to note that this reaction is a gas-solid phase reaction. The system contains only 50 μL of water, which evaporates into water vapor upon injection into the reactor. As a result, only gaseous products such as CH_4_, CO, and a small amount of H_2_ are detected, while liquid products (such as CH_3_OH, HCHO, HCOOH, etc.) are not detected. Figure 3a shows the corresponding photocatalytic CO_2_ performance of the samples under visible-light irradiation. For single metal sulfides In_2_S_3_, Cu_2_S, and SnS_2_, CO is the main product with a yield rate of less than 3.2 μL h^−1^ from photocatalytic CO_2_ reduction, while the multi-electron transfer product CH_4_ is hardly formed. Whereas, the CuInSnS_4_ sample shows excellent photocatalytic CO_2_ reduction performance, yielding CH_4_ as the main product besides a slight amount of CO and H_2_ evolution. The hydrothermal temperature has no obvious impact on the CO_2_ reduction performance of CuInSnS_4_ (Supplementary Fig. 12). The rate of CH_4_ generation reaches 6.53 μL h^−1^ for the CuInSnS_4_ sample. The selectivity of CH_4_ is calculated to be 67.3% based on the contents of carbon-containing products. The significant difference in the product selectivity demonstrates the different mechanisms of CO_2_ reduction or the different active sites between CuInSnS_4_ and single metal sulfides. The controlled blank experiments under other conditions were investigated to confirm the occurrence of CO_2_ reduction on CuInSnS_4_, as shown in Supplementary Fig. 13. The CH_4_ product is not detected without either light irradiation or catalyst, proving that CO_2_ reduction is a light-induced catalytic reaction on CuInSnS_4_. Meanwhile, without adding H_2_O into the reaction system, only a very few products are detected, indicating that H_2_O is also one of the essential reactants involved in the reaction. When N_2_ is used instead of CO_2_ for the reaction, only a small amount of CO is detected, directly proving that the source of CO and CH_4_ products is CO_2_. The presence of small amounts of CO may be attributed to contamination from ambient air, reactors, and equipment, as we can see that small CO products is also detected without CuInSnS_4_ photocatalysts^40^. Figure 3b shows the stability of the CuInSnS_4_ sample for photocatalytic reduction of CO_2_. It is clear that the CuInSnS_4_ sample presents good performance without noticeable activity decrement after three-cycle photocatalytic CO_2_ reduction tests of a total of 27 h (9 h visible-light irradiation for each cycle). Neither crystal structural transformation nor absorption behavior changes are found in the XRD pattern and ultraviolet–visible diffuse reflectance spectra for the CuInSnS_4_ sample after photocatalytic reaction (Supplementary Fig. 14). These results suggest that CuInSnS_4_ possesses good stability during photocatalytic CO_2_ reduction. Moreover, the XPS of the catalyst after the reaction shows that a weak photocorrosion phenomenon occurs in CuInSnS_4_^41,42^ (Supplementary Fig. 15). Specifically, the photogenerated holes or the active oxygen species oxidize the surface S^2−^ of the catalyst to SO_3_^2−^. The peaks with binding energies in the range of 168.26~170.26 eV are assigned to the XPS peaks of SO_3_^2−^ species (Supplementary Fig. 16). The photocatalytic performance of CuInSnS_4_ sample is evaluated under different monochromatic light wavelengths in Fig. 3c. It is observed that as the wavelength of the incident light increases, the photocatalytic activity significantly decreases. However, the CuInSnS_4_ nano-single crystal photocatalyst demonstrates a CH_4_ generation rate of 0.69 μL h^−1^ and CO generation rate of 0.22 μL h^−1^ under the irradiation of 525 nm monochromatic light, which is surprising. The different monochromatic light tests indicate that CuInSnS_4_ is an exceptional catalyst for CO_2_ photoreduction under visible light. The ^13^CO_2_ isotope experiment further validates that CH_4_ product is generated from the photoreduction of CO_2_ molecules, where only ^13^CH_4_ is detected when the reaction is carried out in a ^13^CO_2_ atmosphere, as shown in Fig. 3d. Meanwhile, the ^13^CO_2_ isotope also confirms that the generated CO was indeed a product of CO_2_ photoreduction. When the reaction is conducted in a ^13^CO_2_ atmosphere, the weak peak of ^13^CO with a m/z = 29 was detected due to the low activity of CuInSnS_4_ for CO evolution (Supplementary Fig. 17a). It is noteworthy that the mass spectrum peak at m/z = 28 corresponds to the N_2_ molecule from air interference, as evident from the distinct N_2_ peaks in chromatogram. Additionally, the ^12^CO_2_ experiment further evidences that the generated CO results from the photoreduction of CO_2_ molecules with no peaks at m/z = 29 (Supplementary Fig. 17b).

Although the pristine CuInSnS_4_ only exhibits a yield of CH_4_ evolution of 6.53 μL h^−1^ (corresponding to 5.83 μmol h^−1^ g^−1^) with a selectivity of 67.3%, the activity and selectivity of CH_4_ evolution on CuInSnS_4_ can be improved by coupling with semiconductor photocatalysts or noble metals as cocatalysts. We evaluated the photocatalytic performance of CuInSnS_4_ modified with Pt, CoO, NiO, Co(OH)_2_, and dual co-catalysts Pt and Co(OH)_2_, for the CO_2_ reduction reaction. The composition and chemical states of Pt, CoO, NiO, Co(OH)_2_ cocatalysts are well verified by XRD patterns and XPS spectra (Supplementary Figs. 18–23). Table S2 lists a comparison of the photoreduction activity of CuInSnS_4_ and co-catalyst-modified CuInSnS_4_ photocatalysts, along with the common metal sulfide systems currently used for CO_2_ photoreduction. Clearly, both the yield and selectivity of CH_4_ evolution on CuInSnS_4_ can be significantly improved by modifying with cocatalysts such as Pt, CoO, NiO, and Co(OH)_2_. The activity of CH_4_ evolution on the modified CuInSnS_4_ photocatalysts surpasses the majority of the reported photocatalysts for CO_2_ reduction up to now. Particularly, the incorporation of Co(OH)_2_ as a co-catalyst significantly enhances the CO_2_ photoreduction activity of the CuInSnS_4_ photocatalyst. As the Co(OH)_2_ loading increases, the photoreduction activity of CO_2_ exhibits a characteristic volcanic pattern. With 5%Co(OH)_2_ loading onto CuInSnS_4_, the production rates for CH_4_ and CO, respectively, reach 145.45 and 32.32 μmol h^−1^ g^−1^, corresponding to a CH_4_ selectivity of 81.8%. The generation rates of CH_4_ and CO are 25 times and 13 times that of pure CuInSnS_4_, respectively. Furthermore, when CuInSnS_4_ is modified with a dual co-catalyst of 5% Co(OH)_2_ as an oxidation co-catalyst and 1%Pt as a reduction co-catalyst, CH_4_ production reaches 195.60 μmol h^−1^ g^−1^, along with 22.00 μmol h^−1^ g^−1^ of CO, and a CH_4_ selectivity of 89.9%. Photoelectrochemical characterization was employed to assess the separation efficiency of photogenerated carriers on the modified CuInSnS_4_, as shown in Supplementary Fig. 24. Clearly, the CuInSnS_4_ samples modified with the co-catalyst exhibit a higher photocurrent signal and a smaller electrochemical impedance radius as compared to the parent CuInSnS_4_ sample. The photocurrent increases sequentially in the order of CuInSnS_4_ < 5%Co(OH)_2_/CuInSnS_4_ ≈ 1%Pt/CuInSnS_4_ < 5%Co(OH)_2_/CuInSnS_4_/1%Pt, indicating that the modification of the dual co-catalyst improves photoelectric carrier separation and migration compared to the single co-catalyst. The NiO and CoO cocatalysts also improve the separation efficiency and migration rate of the photogenerated carriers of CuInSnS_4_ photocatalyst. The decreasing order of electrochemical resistance radius is CuInSnS_4_ > 1%Pt/CuInSnS_4_ ≈ 5%Co(OH)_2_/CuInSnS_4_ > 5%Co(OH)_2_/CuInSnS_4_/1%Pt, consistent with the photocatalytic activity trend. Additionally, both 10%NiO/CuInSnS_4_ and 10%CoO/CuInSnS_4_ exhibit smaller electrochemical resistance radii than pure CuInSnS_4_, confirming that the cocatalyst promotes the photogenerated charge separation and migration. Therefore, the modification of CuInSnS_4_ with various cocatalysts to enhance the separation of photogenerated carriers is related to the activity and selectivity of CH_4_ products. The apparent quantum yield is calculated by measuring the yield of CH_4_ and CO in 5%Co(OH)_2_/CuInSnS_4_/1%Pt under monochromatic light at 400 nm. Supplementary Fig. 25 shows the spectrum and intensity of monochromatic light at 400 nm. Under 400 nm monochromatic light irradiation, the apparent quantum efficiencies for CH_4_ and CO are 0.16% and 0.01%, respectively. Based on the above analysis, we believe that CuInSnS_4_ nano-single crystal photocatalysts through further optimization design of the different contents and types of cocatalyst modification can be more efficient and selective for CO_2_ reduction in the future.

### Energy band and photoelectrochemical characterization

**Fig. 4:**
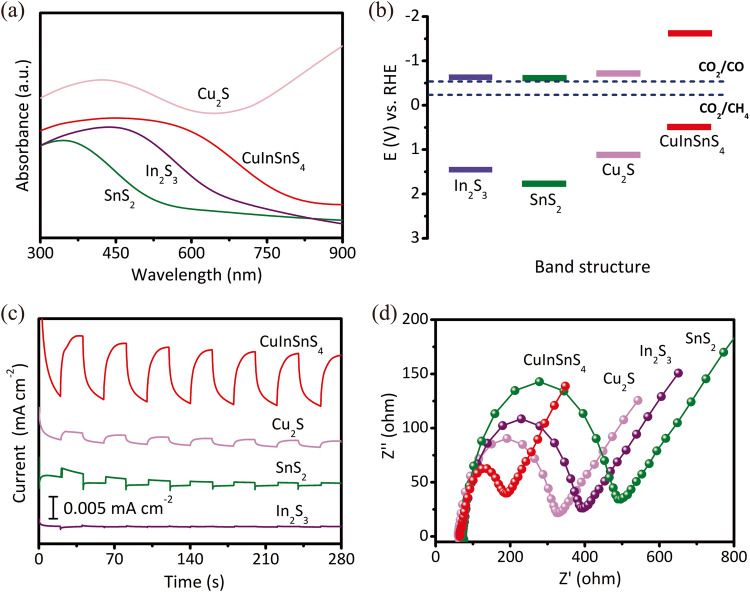
Characterization of energy bands and optoelectronic properties. **a** UV-vis absorption spectrum of various metal sulfides. **b** The optical band gap energy (*E*_g_) of the corresponding CuInSnS_4_ and various single metal sulfides. **c** Photocurrent response and **d** electrochemical impedance spectroscopy of the as-prepared samples.

The band energy potential is a key determinant of the driving force of redox reactions. Therefore, we have studied the band structure of the catalyst through UV-vis DRS and XPS valence band spectroscopy. As shown in Fig. 4a and Supplementary Fig. 26, the optical absorption band edge of CuInSnS_4_ is calculated to be 787.5 nm, which corresponds to a band gap energy of 1.57 eV. For comparison, the absorption band edges of single metal sulfide Cu_2_S, SnS_2_, and In_2_S_3_ are 747.0 nm, 552.7 nm, and 641.6 nm, corresponding to the band gap of 1.66 eV, 2.24 eV, and 1.93 eV, respectively. Moreover, the valence band potential of CuInSnS_4_ is determined to be 0.50 V from the valence band XPS spectra (Supplementary Fig. 27), while the Cu_2_S, SnS_2_, and In_2_S_3_ possess valence band potentials of 1.02 V, 1.70 V, and 1.93 V, respectively. By using the formula *E*_CB_ = *E*_g_–*E*_VB_, we have determined that the conduction band potentials of CuInSnS_4_, Cu_2_S, SnS_2_, and In_2_S_3_ are −1.15 V, −1.10 V, −0.54 V, and −0.55 V, respectively. Based on the optical band gaps, we have obtained the electronic band energies relative to a normal hydrogen electrode (Fig. 4b), indicating that both CuInSnS_4_ and single metal sulfides have the ability to reduce CO_2_ to CH_4_ and CO. Notably, CuInSnS_4_ exhibits the highest reduction potential for the photogenerated electrons to reduce CO_2_. Additionally, CuInSnS_4_ shows a significantly increased photocurrent density (0.014 mA cm^−2^) compared to the single metal sulfide under visible-light irradiation (Fig. 4c), indicating a more efficient separation of the photoinduced charge in multi-metal sulfides. The lower interface resistance in the corresponding electrochemical impedance spectra (Fig. 4d) confirms the rapid transfer of photogenerated electrons in CuInSnS_4_. The efficient separation efficiency and migration rate of photogenerated carriers make polymetallic sulfides exhibit higher photocatalytic CO_2_ reduction performance compared to monometallic sulfides. However, the higher migration and separation efficiency of charge carriers in CuInSnS_4_ alone is not sufficient to explain the substantial difference in the product selectivity between CuInSnS_4_ and single metal sulfides.

### The in situ CO_2_ adsorption FT-IR spectra and mechanism

**Fig. 5:**
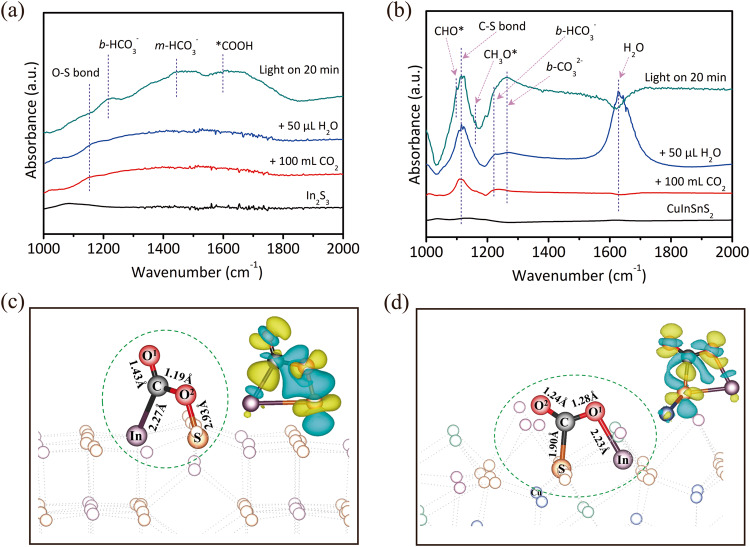
In situ CO_2_ adsorption FT-IR spectra and differential charge density map as well as free energy diagrams of CO_2_ photoreduction to CH_4_. In situ FT-IR spectra of CO_2_ adsorbed on **a** In_2_S_3_ and **b** CuInSnS_4_. CO_2_ adsorption configuration and differential charge density map of **c** In_2_S_3_ and **d** CuSnInS_4_ photocatalysts. In the differential charge density map, the yellow and blue regions indicate electron accumulation and depletion, respectively.

To understand the CO_2_ reduction process over CuInSnS_4_ and single metal sulfides, in situ Fourier-transform infrared spectroscopy is used to compare the reaction intermediates on the catalyst surface. No macroscopic infrared absorption peaks for intermediates are found on Cu_2_S or SnS_2_, even under light irradiation, possibly due to their weak chemical interaction with CO_2_ (Supplementary Fig. 28a, b). However, In_2_S_3_ shows a significant activation effect on the CO_2_ adsorbed on the surface under light irradiation (Fig. 5a). Notably, CO_2_ can form chemical adsorption with In_2_S_3_ even under dark conditions, as indicated by the infrared peak at 1150 cm^−1^, which can be considered an O-S stretching vibration^43^, suggesting that the oxygen atom of CO_2_ is bonded to the sulfur atom of In_2_S_3_. Upon light irradiation, some infrared peaks of the produced intermediates on the catalyst surface are observed. The infrared peak at 1225 cm^−1^ is attributed to the vibration of bidentate bicarbonate^44^, while the infrared peak at 1412 cm^−1^ is attributed to the vibration of monodentate bicarbonate^45^. Most importantly, the infrared peak at 1610 cm^−1^ is attributed to the *COOH group, which is generally regarded as the crucial intermediate for CO_2_ reduction to CO^46^. Surprisingly, the polymetallic sulfide CuInSnS_4_ exhibits strong chemisorption of CO_2_ and strong physisorption of H_2_O (Fig. 5b). The prominent infrared peak observed at 1627 cm^−1^ is attributed to the physical adsorption of H_2_O. However, In_2_S_3_ does not exhibit a noticeable infrared adsorption peak of H_2_O at 1627 cm^−1^. This is because In_2_S_3_ has a lower affinity towards water adsorption than CuInSnS_4_, as indicated by the high contact angle on In_2_S_3_ surface (Supplementary Fig. 29). Moreover, a prominent infrared peak at 1117 cm^−1^ assigned to C-S stretching vibration is observed upon the CO_2_ adsorption on CuInSnS_4_^47–49^. The CO_2_ adsorption and activation are significantly improved on CuInSnS_4_ compared to In_2_S_3_. Furthermore, the adsorption state of CO_2_ on CuInSnS_4_ is different from that on In_2_S_3_, as the carbon atom of CO_2_ is bonded to the sulfur atom of CuInSnS_4_. Under light conditions, CuInSnS_4_ produces specific CO_2_-activated intermediates, as indicated by the infrared peaks at 1225 cm^−1^ and 1260 cm^−1^, attributed to the vibration of bidentate bicarbonate and bidentate carbonate^50,51^, respectively. Additionally, the infrared peaks at 1100 cm^−1^ and 1160 cm^−1^ are attributed to the absorption peaks of *CHO and *CH_3_O, which are intermediates for the yield of CH_4_^52,53^. Therefore, either the CO_2_ adsorption state in darkness or the produced intermediates under light irradiation show that the sites for CO_2_ adsorption and the CO_2_ reduction approach differ between single-metal sulfide In_2_S_3_ and multi-metal sulfide CuInSnS_4_. This may account for the different selectivity of products between In_2_S_3_ and CuInSnS_4_.

It has been demonstrated that sulfur defect sites in multiple metal sulfides acted as an active center for the selective photoreduction of CO_2_ to CH_4_. However, CuInSnS_4_ nano-single crystal shows no significant sulfur defect signals compared to the strong defect signals in SnS_2_ and In_2_S_3_, as shown in Supplementary Fig. 30. This indicates that the selectivity of CH_4_ products on CuInSnS_4_ is not related to sulfur defects. The mechanism of selective photocatalytic CO_2_ reduction on CuInSnS_4_ and In_2_S_3_ photocatalysts is further theoretically studied. Firstly, we investigated the adsorption behavior of CO_2_ on the surfaces of In_2_S_3_ and CuInSnS_4_. The (0 0 1) crystal plane of the In_2_S_3_ sample and the (1 1 1) crystal plane of the CuInSnS_4_ sample were selected as models, and all atoms on the crystal planes were considered as potential sites for CO_2_ adsorption activation (Supplementary Figs. 31 and  32). Based on CO_2_ adsorption energy, the optimal adsorption models of CO_2_ on In_2_S_3_ and CuInSnS_4_ photocatalyst surfaces are optimized (Supplementary Figs. 33 and  34). Figure 5c, d depict schematic diagrams of CO_2_ stable adsorption configurations and the charge density difference of CO_2_ on In_2_S_3_ and CuInSnS_4_, respectively. The stable CO_2_ adsorption configuration on In_2_S_3_ is the C atom of CO_2_ bonded to In atom with a bond length of 2.27 Å, while the O atom of the CO_2_ molecule is bonded with the S atom with a bond length of 1.70 Å. The CO_2_ adsorption model for the polymetallic sulfide CuInSnS_4_ is the opposite. The unique C-S bond with a bond length of 1.90 Å is formed between the C atom of CO_2_ and the surface S atom, and one O atom of CO_2_ is bonded with In atom with a bond length of 2.23 Å. The various adsorption configurations of CO_2_ on In_2_S_3_ and CuInSnS_4_ surfaces are attributed to the coordination environment and charge number of the surface S atoms. The surface S atom of In_2_S_3_ is an electron-rich site with [SIn_3_] coordination structure, while the S atom on CuInSnS_4_ is an electron-poor center with [SInSnCu] coordination structure (Supplementary Fig. 35 and Table S1). Different adsorption configurations may be the key to determining the direction of electron transfer and thus the selectivity of CO_2_ reduction on CuInSnS_4_ and In_2_S_3_. Adsorption of CO_2_ on the In_2_S_3_ (0 0 1) crystal plane leads to inconsistent changes in two C=O lengths. The length of the C-O^**2**^ bond is 1.43 Å, equal to the ordinary C-O (1.43 Å) single bond, while the length of C-O^**1**^ is shortened to 1.19 Å, close to the length of C-O (1.12 Å) in a CO molecule. This asymmetric activation is more likely to cause the rupture of C-O^**2**^, thereby preferentially producing CO on In_2_S_3_. In the case of the CuInSnS_4_ (1 1 1) crystal plane, both C-O bonds are similar in length, measuring 1.26 ± 0.02 Å. They are longer than the C-O bond (1.16 Å) in a free CO_2_ molecule, indicating that the bond energy of two C=O bonds of the activated CO_2_ is simultaneously weakened. The calculation of the charge density difference reveals the difference in electronic structure and electron flow resulting from the interaction of CO_2_ with the surface atoms of In_2_S_3_ and CuInSnS_4_. On the In_2_S_3_ surface, there is extensive charge depletion for the C-O^**2**^ and S–O^**2**^ bonds, which implies that these chemical bonds are weakened. In contrast, CO_2_ exhibits a wide charge accumulation region on CuInSnS_4_, leading to the formation of a strong C-S and In-O chemical bond. This strong interaction is beneficial for the firm adsorption of CO_2_ on the CuInSnS_4_ surface, promoting the further deep reduction reaction. The Bader charges analysis further confirms that there is more charge transfer between the CuInSnS_4_ surface and CO_2_ molecules adsorbed on it. The surface of In_2_S_3_ and CuInSnS_4_ loses 0.28e and 0.32e, respectively, after CO_2_ adsorption (Table S3).

**Fig. 6:**
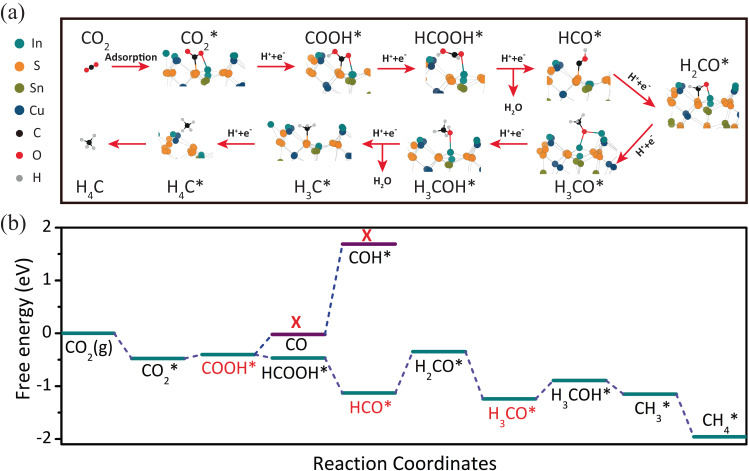
DFT calculations of the CO_2_ conversion pathway. **a** Calculated adsorption configuration of CO_2_ and reactive intermediates on CuSnInS_4_. **b** Gibbs free energy diagrams for CO_2_ reduction to CH_4_ on CuSnInS_4_.

The stable configuration of CO_2_ adsorption on CuInSnS_4_ determines its excellent CO_2_ photoreduction activity and selectivity. Therefore, DFT calculations were further carried out to study the conversion pathway of CO_2_ on the CuInSnS_4_ photocatalyst surface, as shown in Fig. 6. In Fig. 6a, the adsorption configuration of CuInSnS_4_ is shown for each intermediate step, from CO_2_ adsorption to CH_4_ generation. The C atoms of various intermediates, such as CO_2_*, COOH*, CHO*, CH_2_O*, and CH_3_*, remain in a stable bond to the electron-deficient S atoms of the CuInSnS_4_ nano-single crystal (1 1 1) plane. With the addition of protons and electrons, the removal of the H_2_O molecule results in the breaking of the chemical bond between O atoms of intermediates and In atoms on CuInSnS_4_. Moreover, unlike most metal sulfide photocatalysts, the hydrogenation of CO_2_ adsorbed on the CuInSnS_4_ surface to COOH* is an easy step, requiring only a potential energy barrier of 0.075 eV^33^, as shown in Fig. 6b. This is attributed to the fact that the electrons are localized on the O^**2**^ atom of CO_2_ molecules in the S-C-O-In adsorption configuration of CO_2_ on the CuInSnS_4_ surface to facilitate the addition of protons, thus lowering the formation energy of COOH* intermediates. However, converting COOH* to CO is an endothermic reaction that must overcome an energy barrier of 0.46 eV. On the other hand, the continuous hydrogenation of COOH* intermediates to produce HCOOH* is an exothermic reaction, promoting the hydrogenation of CO_2_. The formation of H_2_CO* intermediates is the rate-limiting step for further hydrogenation processes, but the hydrogenation of H_2_CO* to H_3_CO* and finally to CH_4_ is thermodynamically spontaneous. Therefore, CuInSnS_4_ can achieve high selectivity for CH_4_ products. Additionally, the adsorption energies of each intermediate product can explain the high CH_4_ selectivity. Supplementary Fig. 36 shows that the adsorption energies of CH_4_, CH_3_OH, and HCOOH on the CuInSnS_4_ surface are −0.17, −0.61, and −0.67 eV, respectively, with CH_4_ having the highest adsorption energy. This indicates that CH_4_ products are most easily desorbed from the CuInSnS_4_ surface, which is one of the reasons why the CuInSnS_4_ photocatalyst has high selectivity for the photoreduction of CO_2_ into CH_4_.

**Fig. 7:**
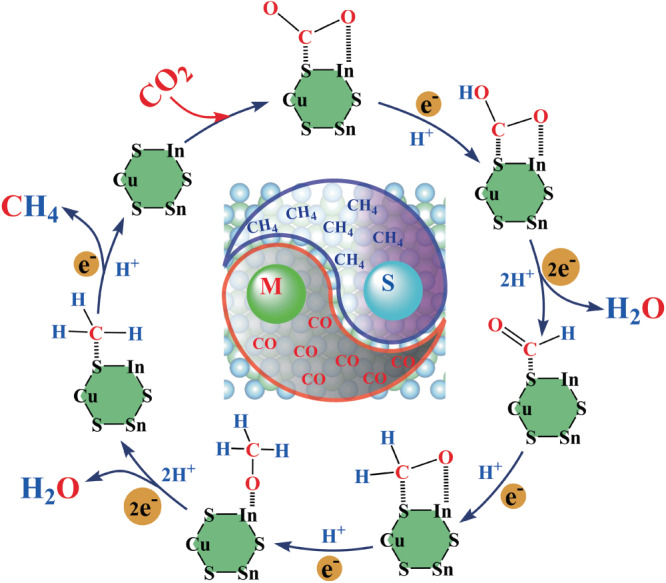
CO_2_ photoreduction pathway. Proposed photocatalytic mechanism for CO_2_ reduction on the CuInSnS_4_. The backgroup crystal structure was created by VESTA program^56^.

The photoreduction mechanism of CO_2_ on the CuSnInS_4_ surface is proposed in Fig. 7. The first step involves CO_2_ adsorbing on the catalyst surface to form the unique S-C-O-In structural unit. This process weakens the C=O double bond in the CO_2_ molecule while non-metallic S atoms serve as adsorption sites, ensuring a strong bond to the C atom of CO_2_. This benefits the continuous reduction of CO_2_ molecules into COOH*, HCO*, H_3_CO* intermediates, and ultimately into CH_4_ on the catalyst surface through the assistance of photogenerated electrons and protons. Lastly, the low adsorption energy of CH_4_ on the catalyst surface facilitates its quick release, completing the full photocatalytic cycle reaction.

In summary, a CuSnInS_4_ nano-single-crystal photocatalyst with exposed (1 1 1) facets is successfully prepared by a simple one-step hydrothermal reaction. Under visible-light irradiation, the CuSnInS_4_ nano-single crystal photocatalyzes the conversion of CO_2_ and H_2_O into main products of CH_4_ with a generation rate of 6.53 μL h^−1^, significantly higher than that of single metal sulfides (In_2_S_3_, Cu_2_S, and SnS_2_). The electron-poor center sulfur atom on the CuSnInS_4_ (1 1 1) crystal plane acts as the site for CO_2_ adsorption and activation, which leads to the activation of the two symmetrical C=O double bonds of CO_2_ molecule to form a stable S-C-O-In transition state. This induces CH_4_ generation via the conversion route of COOH*→HCOOH*→H_2_CO*→H_3_CO*→CH_4_*. However, the asymmetric activation of CO_2_ by monometallic sulfides is more likely to result in the cleavage of individual C-O bonds in the CO_2_ molecule, leading to the preferential photoreduction of CO_2_ to CO. This work provides a distinctive understanding of catalysts for CO_2_ adsorption and activation for the CO_2_ selective conversion to help the conversion of CO_2_ resources into high-value-added products.

## Methods

### Preparation of CuInSnS_4_ nanocrystal

A simple one-step hydrothermal method was used to synthesize CuInSnS_4_ nanocrystal photocatalyst with the cubic crystal structure. The detailed operation process is as follows. Firstly, 1 mmol of CuCl, 1 mmol of SnCI_4_·5H_2_O, and 1 mmol of InCI_3_·4H_2_O were added to 40 mL of deionized water to form a solution under vigorous stirring. Then, 5 mmol of TAA was dissolved in the above-mixed solution and stirred at room temperature for 30 min. Finally, the resulting mixed solution was transferred to a 50 mL Teflon-lined autoclave and sealed into a stainless steel tank for hydrothermal reaction. The hydrothermal temperature is controlled at 160, 180, and 200 °C for 24 hours. After the reaction, the product was collected and washed with deionized water, and dried under vacuum at 60 °C. The obtained samples were labeled CuInSnS_4_ (160 °C), CuInSnS_4_ (180 °C), and CuInSnS_4_ (200 °C) according to the reaction temperature. The detailed preparation processes of CuInSnS_4_ modified Pt, CoO, NiO, and Co(OH)_2_ cocatalysts can be seen in supplementary information.

### Characterization

X-ray diffractometer (D8 Advance, Bruker) was used to analyze the crystal structure of the catalyst. The XRD test range is 10°−80°, and the scan rate is 10° min^−1^. Scanning electron microscopy (su8010, Hitachi) was used to observe the surface morphology of the catalyst. The element composition and ratio of the sample are detected by EDS. The apparent morphology and high-resolution TEM image of the catalyst were tested by transmission electron microscope (TEM, TECNAI G2F20, FEI Company). At the same time, SAED and element mapping images of the catalyst were obtained in the TEM measurement mode. A UV-VIS-NIR Spectrophotometer (Cary 500) was used to obtain the catalyst UV-VIS-NIR DRS, in which BaSO_4_ was used as a standard sample for 100% light transmission. The Micromeritics 3020 M physical adsorption instrument was used to obtain the nitrogen adsorption and desorption curves of different catalysts. The specific surface area of each catalyst was calculated from the type of nitrogen adsorption and desorption curves. The catalyst and dried potassium bromide were evenly ground, and 20 mg was weighed and pressed into slices, then placed in a quartz infrared tube for a carbon dioxide adsorption infrared test. In situ infrared spectra measurements were performed using a Fourier-transform infrared spectrometer (Nicolet iS50 FT-IR Spectrometer) equipped with a mercury cadmium telluride detector (Supplementary Fig. 37). In situ infrared spectra were recorded by averaging 32 scans at a resolution of 4 cm^−1^. To initiate the experiment, the catalyst was placed in a 250 mL quartz tube and compacted into a film. The tube was then subjected to vacuum treatment for 60 min. Subsequently, high-purity CO_2_ gas was introduced, and the quartz tube was sealed. A liquid sampler was used to inject 60 μL of deionized water into the sealed quartz tube. The tube was heated with a hot blower to vaporize the deionized water. The quartz tube was positioned in the FT-IR spectrometer, ensuring that the incident light of the infrared spectrometer was perpendicular to the sample surface. A xenon lamp visible-light source was introduced to directly illuminate the sample surface. Infrared spectra were recorded after pretreating the catalyst in a vacuum, introducing CO_2_ gas, and vaporizing deionized water, respectively. After the introduction of light, infrared spectra were recorded every 5 min. Gas chromatography-mass spectrometry (Agilent 7890B, Agilent 5977B MSD) was used to analyze ^13^CH_4_ and ^13^CO. Electron paramagnetic resonance spectroscopic measurements were performed at room temperature using a Bruker A300 EPR spectrometer.

### Photocatalytic performance

300 W xenon lamp (Microsolar 300, Beijing Perfectlight Technology Co., Ltd.) was equipped with a 420 nm cutoff wavelength filter as a light source that simulates visible light for photocatalytic CO_2_ reduction tests. Firstly, 50 mg of catalyst was dispersed in 5 mL of deionized water and sonicated for 10 min. Then the catalyst was dropped into a watch glass with a diameter of 3 cm and dried at 80 °C. Subsequently, the dried catalyst was placed in a quartz reactor with a volume of ~250 cm^3^, and then high-purity CO_2_ gas (99.999%) was introduced to replace all the air. The flow rate of carbon dioxide gas was ~100 mL/min and lasted for 1 h. Finally, 50 μL deionized water was injected into the quartz reactor from the rubber stopper through a gas chromatography liquid syringe (maximum range, 50 μL), and the reactor was heated with a hair dryer to evaporate the water into water vapor. As a result, water is present in the form of water vapor throughout the reaction. The reactor was placed under a xenon lamp for photocatalytic reaction, and the current of the xenon lamp was 16 A. After 1 h of illumination, 0.5 mL of gas was extracted from the reactor with a 1 mL gas chromatograph gas syringe and injected into the gas chromatograph for product analysis and detection. Among them, H_2_, O_2_, and N_2_ are detected by a thermal conductivity detector. CH_4_ was detected by the flame ionization detector. CO passes through the flame ionization detector after being transformed by the nickel reformer. The product was qualitatively and quantitatively analyzed by gas chromatography retention time and appearance standard curve method.

### Theoretical calculations

The density functional theory calculations were performed using the VASP code with the projected augmented wave method^54^. Generalized gradient approximation in the scheme of Perdew-Bueke-Ernzerhof was used for the exchange-correlation functional^54^. The PBEsol exchange-correlation function was adopted in the optimization calculations. Grimme’s DFT-D3 scheme was used to describe the long-range vdW interactions^54^. The cutoff energy of the basis function was 420 eV. For the CuSnInS_4_ (1 1 1) crystal plane, a 2 × 2 × 1 supercell with a four-layer slab was constructed, and only the top two layers were allowed to relax. A vacuum region of 12 Å was set above the surface for periodic boundary conditions, and dipole correction was also applied. Gamma-centered 1 × 1 × 1 grid k-points were used for the interface. Geometry relaxation was performed until the energy, and atomic forces converged to be smaller than 10^−5 ^eV and 0.03 eV/Å. Charge transfers were calculated using the Bader charge analysis^54,55^.

The free energy of each reaction intermediate was determined by: G = E + ZPE–TS. The electronic energy was directly obtained from DFT calculations. The zero-point energy and entropy correction (TS, T = 298.15 K) were computed from vibration analysis according to standard methods. The adsorption-free energy of the adsorbates can be calculated using: ∆Gads = ∆Eads + ∆ZPE–T∆S, where ∆Eads is the adsorption energy of the adsorbates, and ∆ZPE and ∆S are the difference between ZPE and S, respectively. After the adsorption-free energies of the adsorbates are obtained, the reaction-free energies of CO_2_ reduction reaction elementary steps can be determined correspondingly by using the computational hydrogen electrode model^40^.

### Supplementary information

Supplementary Information

Peer Review File

### Source data

Source data

## Data Availability

The authors declare that the data supporting the findings of this study are available in the paper and its supplementary information files. Source data are provided with this paper.
