# Supplementary material for: Metal to non-metal sites of metallic sulfides switching products from CO to CH4 for photocatalytic CO2 reduction
Source: Nat Commun. 2023 Oct 4;14:6168. doi: 10.1038/s41467-023-41943-x (PMC10550947; doi:10.1038/s41467-023-41943-x)
Supplement: Supplementary file 1 — Supplementary Information [file 41467_2023_41943_MOESM1_ESM.pdf]

Supplementary Information

Metal to non-metal sites of metallic sulfides switching products from CO to CH_4 for photocatalytic CO_2 reduction

Yao Chai[1,‡], Yuehua Kong[2,‡], Min Lin[1], Wei Lin[2], Jinni Shen[1], Jinlin Long[1], Rusheng Yuan[1], Wenxin Dai[1,3], Xuxu Wang[1], Zizhong Zhang[1,3,*]

[1]State Key Lab of Photocatalysis on Energy and Environment, College of Chemistry, Fuzhou University, Fuzhou, P. R. China

[2]College of Chemistry, Fuzhou University, Fuzhou, P. R. China

[3]Qingyuan Innovation Laboratory, Quanzhou, P. R. China

‡These authors contributed equally

*Corresponding author E-mail: z.zhang@fzu.edu.cn

Contents

Supplementary Notes……S1
Supplementary Methods……S1
Supplementary Figures and Tables……S4
Supplementary Fig. 1 XRD patterns of different $CuInSnS_4$ samples……S4
Supplementary Fig. 2 XRD patterns of In_2S_3, SnS_2, and Cu_2S……S4
Supplementary Fig. 3 Schematic diagram of the crystal structure of $CuInSnS_4$…S5
Supplementary Fig. 4 Schematic diagram of the crystal structure of In_2S_3……S5
Supplementary Fig. 5 Schematic diagram of the crystal structure of Cu_2S……S6
Supplementary Fig. 6 Schematic diagram of the crystal structure of SnS_2……S6
Supplementary Fig. 7 SEM images of In_2S_3, Cu_2S, and SnS_2……S7
Supplementary Fig. 8 SEM image of $CuInSnS_4$……S7
Supplementary Fig. 9 N_2 adsorption and desorption curves of Cu_2S, In_2S_3, $CuInSnS_4$ and SnS_2……S8
Supplementary Fig. 10 Cu LMM spectra of $CuInSnS_4$……S9
Supplementary Fig. 11 Xenon light source spectrum and light intensity……S9
Supplementary Fig. 12 Photocatalytic CO_2 reduction performance of a series of $CuInSnS_4$ photocatalysts under different preparation temperatures……S10
Supplementary Fig. 13 The $CuInSnS_4$ sample photoreduced CO_2 activity under different test conditions……S10
Supplementary Fig. 14 XRD and UV-Vis DRS of sample before and after the reaction……S11
Supplementary Fig. 15 XPS spectra of $CuInSnS_4$ (180°C) before and after the reaction……S12
Supplementary Fig. 16 O*1s* spectra of $CuInSnS_4$ (180°C) photocatalyst before and after reaction……S14
Supplementary Fig. 17 GC-MS spectra of ^{13}CO and ^{12}CO……S15
Supplementary Fig. 18 XRD patterns of 5%$Co(OH)_2$/$CuInSnS_4$, 5%$Co(OH)_2$/$CuInSnS_4$/1%Pt, 10%CoO/$CuInSnS_4$, and 10%NiO/$CuInSnS_4$……S16
Supplementary Fig. 19 Co*2p* and O*1s* XPS spectra of 5%$Co(OH)_2$/$CuInSnS_4$…S17
Supplementary Fig. 20 Ni*2p* and O*1s* XPS spectra of 10%NiO/$CuInSnS_4$……S18
Supplementary Fig. 21 Co*2p* and O*1s* XPS spectra of 10%CoO/$CuInSnS_4$……S19
Supplementary Fig. 22 Pt*4f* XPS spectra of 1%Pt/$CuInSnS_4$……S20
Supplementary Fig. 23 Pt*4f* and Co*2p* XPS spectra of 5%$Co(OH)_2$/$CuInSnS_4$/1%Pt sample……S21
Supplementary Fig. 24 Photocurrent response and electrochemical impedance spectroscopy of the as-prepared samples……S22
Supplementary Fig. 25 Spectrum and intensity of 400 nm monochromatic light…S22
Supplementary Fig. 26 The optical band gap energy of the corresponding $CuInSnS_4$ and various single metal sulfides……S23
Supplementary Fig. 27 Valence-band XPS spectra of the $CuInSnS_4$ and various single metal sulfides……S24

Supplementary Fig. 28 In situ FT-IR spectra of CO_2 adsorbed on different photocatalysts……S24
Supplementary Fig. 29 Contact angles of $CuInSnS_4$ and In_2S_3……S25
Supplementary Fig. 30 EPR spectra of various metal sulfides……S25
Supplementary Fig. 31 Theoretically designed adsorption configuration of CO_2 on the In_2S_3 surface……S26
Supplementary Fig. 32 Theoretically designed adsorption configuration of CO_2 on the $CuInSnS_4$ surface……S27
Supplementary Fig. 33 Theoretically designed adsorption configuration of CO_2 molecules on the In_2S_3 surface and the corresponding adsorption configuration after optimization……S29
Supplementary Fig. 34 Theoretically designed adsorption configuration of CO_2 on the $CuInSnS_4$ surface……S30
Supplementary Fig. 35 Coordination mode of S atom on the surface of In_2S_3 and $CuInSnS_4$……S31
Supplementary Fig. 36 The adsorption model of CH_4, CH_3OH and HCOOH on the surface of $CuInSnS_4$ photocatalyst……S32
Supplementary Fig. 37 In situ infrared testing device……S33
Table S1 ICP-MS test of each element of $CuInSnS_4$ sample……S33
Table S2 Comparing the photocatalytic CO_2 reduction performance of $CuInSnS_4$, modified $CuInSnS_4$, and common photocatalysts……S34
Table S3 Quantify the electron transfer between the surface and CO_2……S36
Supplementary References……S37

1. Supplementary Notes.

Raw Materials. Cuprous chloride (CuCl, 99.999%), Indium chloride tetrahydrate ($InCl_3 \cdot 4H_2O$, 99.9%), Cobalt nitrate hexahydrate [$Co(NO_3)_2 \cdot 6H_2O$, 99.999%], Nickel nitrate hexahydrate [$Ni(NO_3)_2 \cdot 6H_2O$, 99.999%], Chloroplatinic acid hexahydrate ($H_2PtCl_6 \cdot 6H_2O$, AR, Pt ≥37.5%), Sodium hydroxide (NaOH, 98%) were purchased from Aladdin Reagent Company (Shanghai, China). Tin chloride pentahydrate ($SnCl_4 \cdot 5H_2O$, 99%), and Thioacetamide (TAA, 99%) were purchased from Macklin Reagent Company (Shanghai, China). Chemicals are used directly without any purification treatment.

2. Supplementary Methods.

Preparation of In_2S_3 Nanosheets. 2 mmol of $InCl_3 \cdot 4H_2O$ and 4 mmol of TAA were dissolved in 40 mL of deionized water and reacted under hydrothermal conditions of 180°C for 12 hours. After the reaction, the product was collected and washed with deionized water, and dried under vacuum at 60°C.

Preparation of SnS_2 Nanosheets. 2 mmol of $SnCl_4 \cdot 5H_2O$ and 5 mmol of TAA were dissolved in 40 mL of deionized water and reacted under hydrothermal conditions of 180°C for 8 hours. After the reaction, the product was collected and washed with deionized water, and dried under vacuum at 60°C.

Preparation of Cu_2S Nanoparticles. 2 mmol CuCl and 3 mmol TAA were dispersed in 40 mL deionized water and reacted under hydrothermal conditions of 180°C for 24 hours. After the reaction, the product was collected and washed with deionized water, and dried under vacuum at 60°C.

Preparation of 1%Pt/$CuInSnS_4$ Photocatalyst. The Pt modified photocatalyst was prepared by the photodeposition method. In detail, 200 mg of $CuInSnS_4$ was firstly dispersed in a mixed solution of 150 mL H_2O and 20 mL CH_3OH. And then, 530 μL $H_2PtCl_6·6H_2O$ solution (10 mg mL^{-1}) was added to the above dispersion liquid. The reaction solution system was evacuated and stirred for 1 hour. Subsequently, visible light was irradiated for 1 hour to reduce $H_2PtCl_6·6H_2O$ to Pt on $CuInSnS_4$ surface. Finally, the catalyst samples were washed with deionized water by centrifugation and dried under vacuum at 60°C to obtain 1%Pt/$CuInSnS_4$ photocatalyst.

Preparation of 10%CoO or 10%NiO modified $CuInSnS_4$ photocatalyst. 50 mg $CuInSnS_4$ was dispersed in 10 mL of H_2O, and then 6.0 mg $Co(NO_3)_2·6H_2O$ or 19.5 mg of $Ni(NO_3)_2·6H_2O$ was added for 3 hour stirring. The H_2O was evaporated at 90°C in an oven. The resulting powder was ground and transferred to a tube furnace for heating treatment at 350 °C for 1 hour with a heating rate of 2 °C min^{-1} under inert Ar gas atmosphere. After cooling to room temperature, the catalyst was washed several times with deionized water and dried under vacuum at 60°C.

Preparation of $Co(OH)_2$/$CuInSnS_4$ Photocatalyst. The $Co(OH)_2$ modified photocatalyst was prepared by the precipitation method. 100 mg of $CuInSnS_4$ was dispersed in 50 mL of deionized water and stirred evenly. Subsequently, a certain amount of $Co(NO_3)_2·6H_2O$ solution (1 mg·mL^{-1}) was added, and stirring was continued for 12 hours. Finally, 10 mL of NaOH solution (2 mg mL^{-1}) was added, and stirring was continued for an additional 12 hours. After the reaction, the samples were washed several times with deionized water and dried under vacuum. The amounts of $Co(NO_3)_2·6H_2O$ solution addition are 9.39, 15.66, 25.06 and 31.32

mL, corresponding to 3%, 5%, 8% and 10% $Co(OH)_2$ loading to $CuInSnS_4$, respectively.

Preparation of 5%$Co(OH)_2$/$CuInSnS_4$/1%Pt Photocatalyst. The $Co(OH)_2$/$CuInSnS_4$/Pt photocatalyst was prepared in two steps. Firstly, 1%Pt was deposited on the surface of the $CuInSnS_4$ sample through photodeposition. Subsequently, 5%$Co(OH)_2$ was loaded on the surface of Pt/$CuInSnS_4$ by the precipitation method.

Supplementary Photoelectrochemical Measurements. The preparation of the working electrode was as follows. 30 mg photocatalyst was dispersed in 5 mL of C_2H_5OH solution and sonicated for 30 min. Then using a 100 μL pipette to suck 60 μL mixed liquid was dripped onto the conductive glass and dry at 60°C. Finally, nail polish was applied to the parts except for covering the catalyst film to avoid the photo-current signal generated by the conductive glass itself. On the SP-150 electrochemical workstation (Bio-Logic, French), a three-electrode system was used for electrochemical testing. Among them, the Ag/AgCl electrode was used as the reference electrode, the platinum foil was used as the counter electrode, and a FTO glass electrode covered by thin-film catalyst film as a working electrode. 0.2 M Na_2SO_4 was used as electrolyte for photocurrent test. The light source was the same as the light source used in the CO_2 reduction experiment. The electrochemical impedance spectroscopy measurement was to choose a mixed solution of 0.5 M KCl and 5.0 mM $K_3[Fe(CN)_6]/K_4[Fe(CN)_6]$ as the electrolyte.

3. Supplementary Figures and Tables.

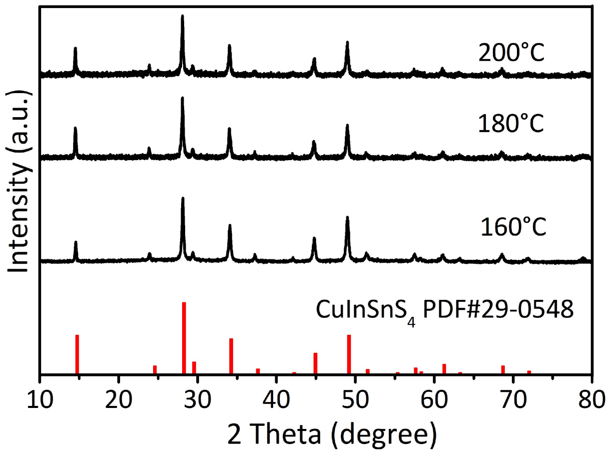

Supplementary Fig. 1 XRD patterns of $CuInSnS_4$ samples obtained under different preparation temperature conditions.

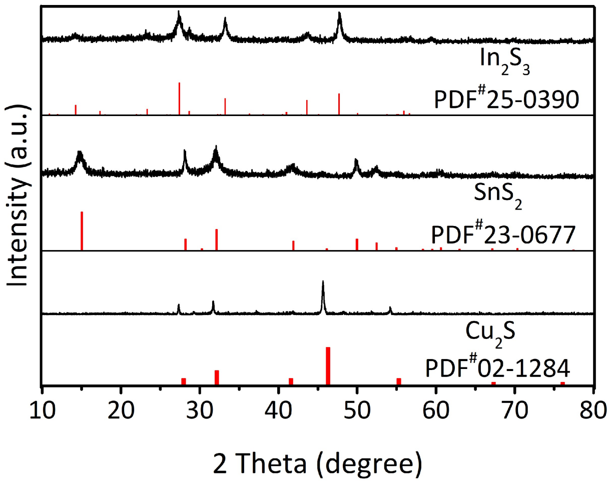

Supplementary Fig. 2 XRD patterns of In_2S_3, SnS_2, and Cu_2S.

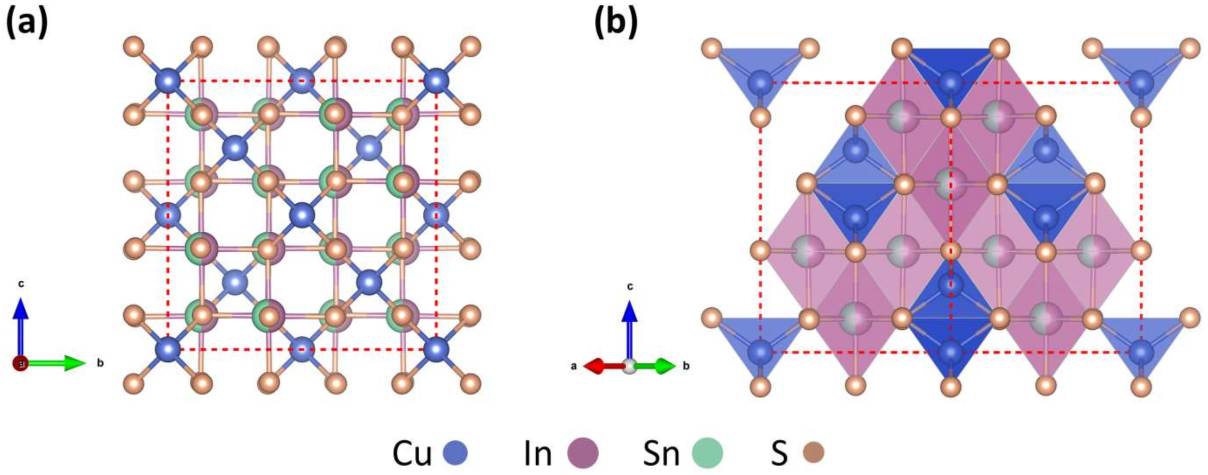

Supplementary Fig. 3 Schematic diagram of the crystal structure of $CuInSnS_4$. (a) Ball-and-stick models. (b) Polyhedral models. The VESTA programme was used for visualising the crystal structures[1].

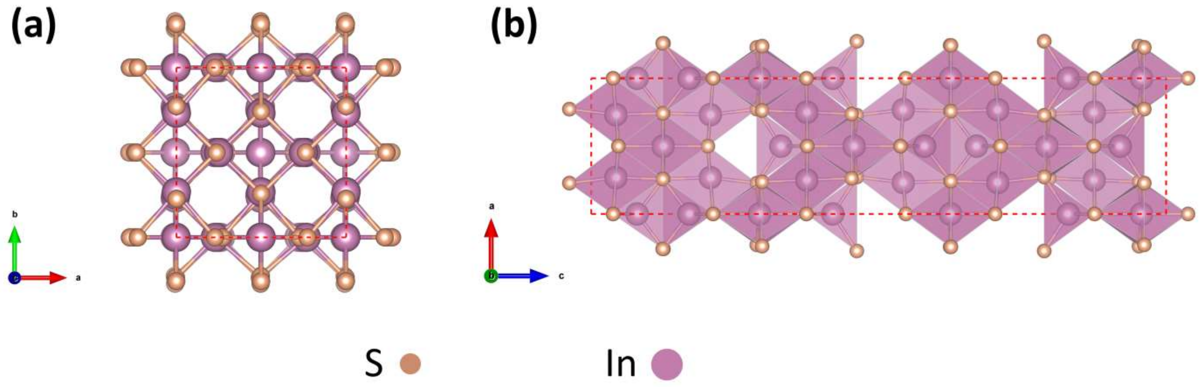

Supplementary Fig. 4 Schematic diagram of the crystal structure of In_2S_3. (a) Ball-and-stick models. (b) Polyhedral models. The VESTA programme was used for visualising the crystal structures[1].

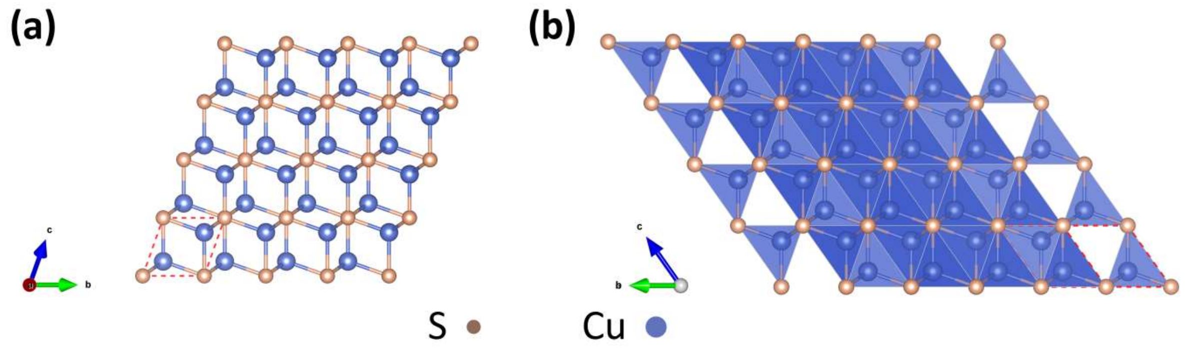

Supplementary Fig. 5 Schematic diagram of the crystal structure of Cu_2S. (a) Ball-and-stick models. (b) Polyhedral models. The VESTA programme was used for visualising the crystal structures[1].

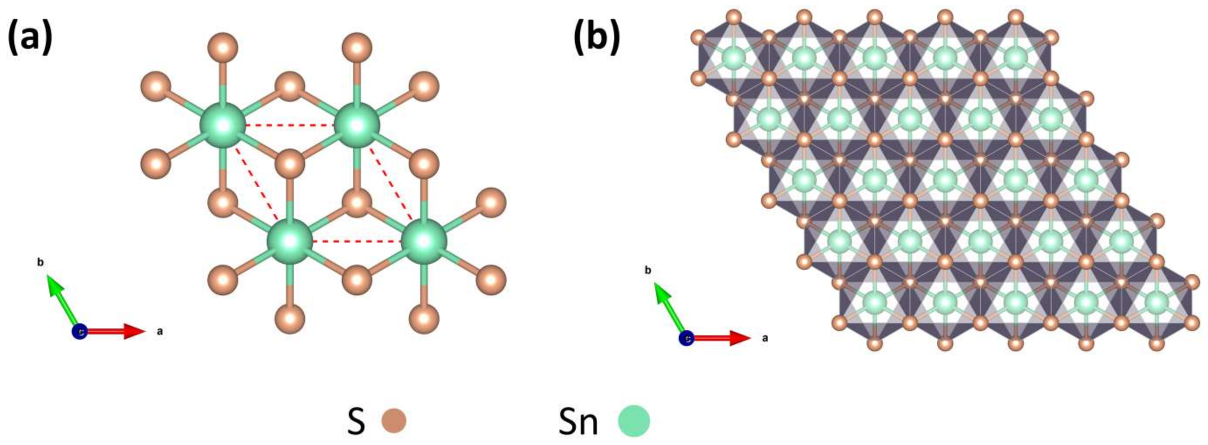

Supplementary Fig. 6 Schematic diagram of the crystal structure of SnS_2. (a) Ball-and-stick models. (b) Polyhedral models. The VESTA programme was used for visualising the crystal structures[1].

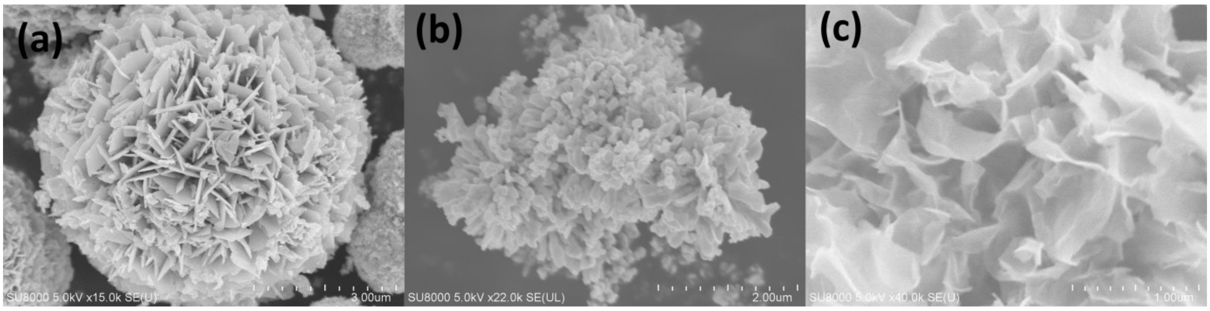

Supplementary Fig. 7 SEM images of (a) In_2S_3, (b) Cu_2S, and (c) SnS_2.

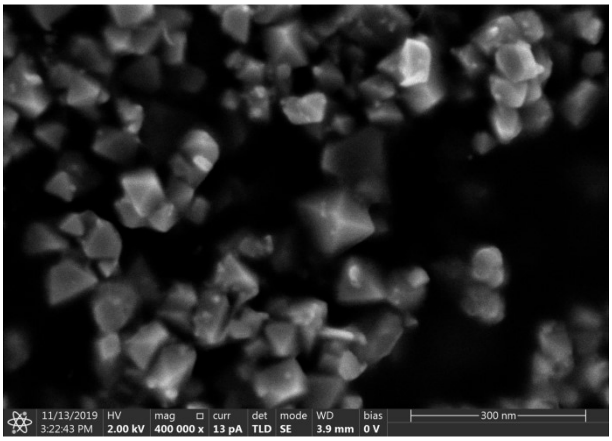

Supplementary Fig. 8 SEM image of $CuInSnS_4$ (180°C) sample.

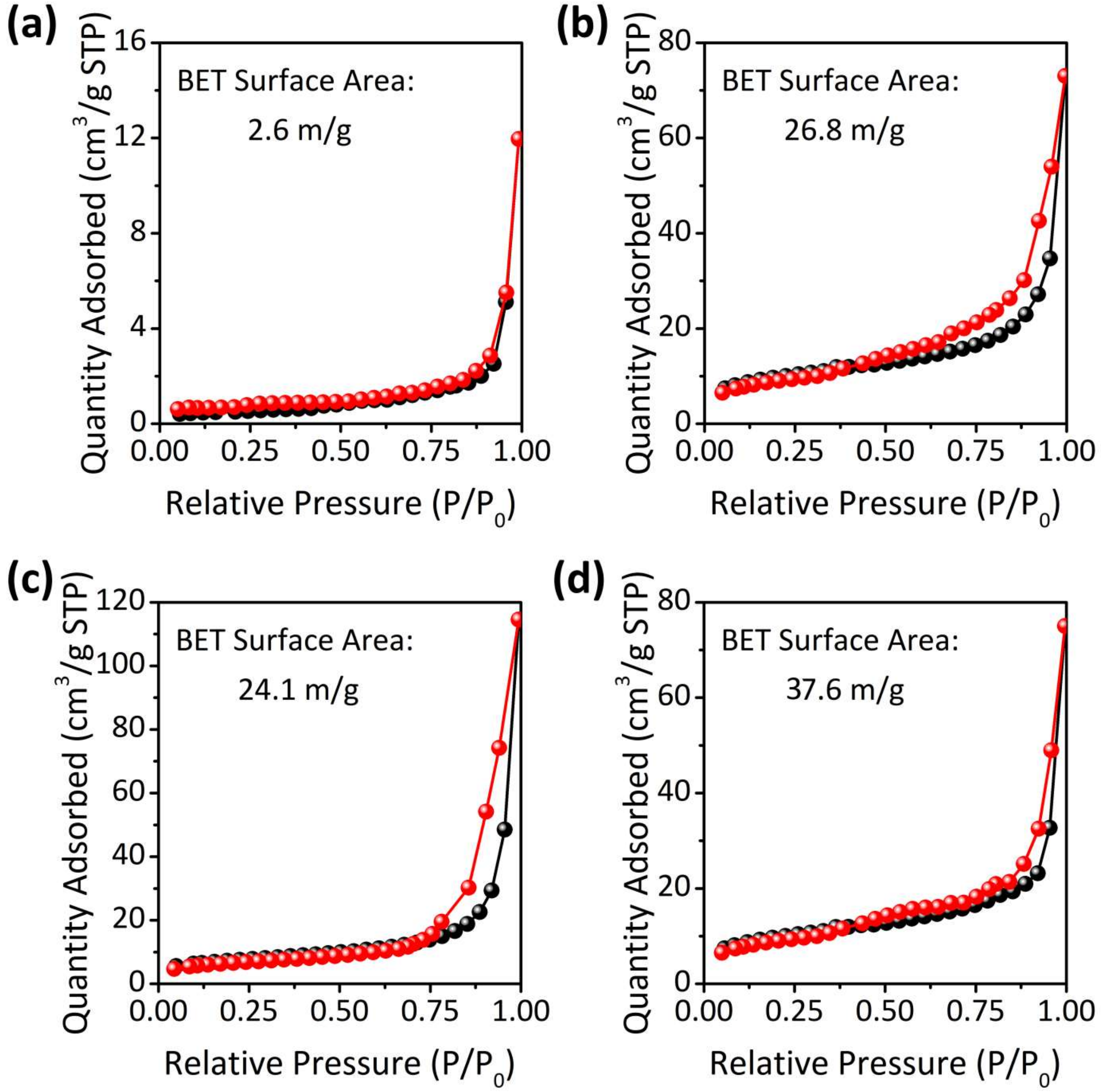

Supplementary Fig. 9 N_2 adsorption and desorption curves of (a) Cu_2S, (b) In_2S_3, (c) $CuInSnS_4$ (180°C), and (d) SnS_2.

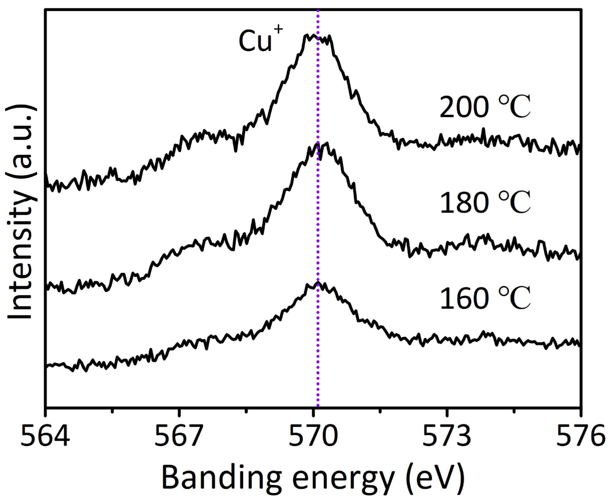

Supplementary Fig. 10 Cu LMM spectra of $CuInSnS_4$ (180°C).

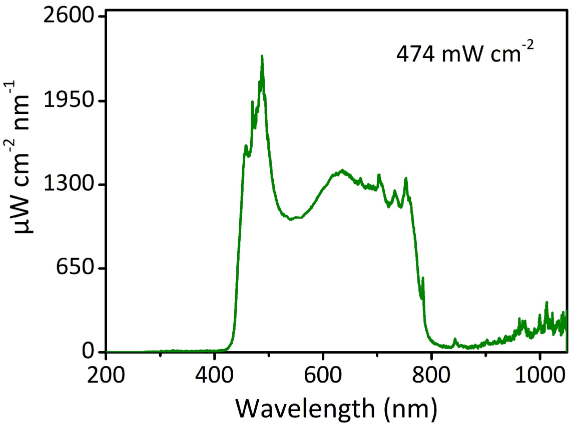

Supplementary Fig. 11. Xenon light source spectrum and light intensity.

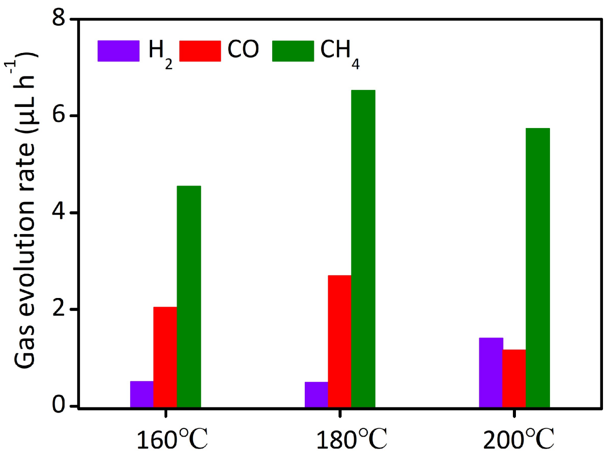

Supplementary Fig. 12 Photocatalytic CO_2 reduction performance of a series of $CuInSnS_4$ photocatalysts under different preparation temperatures.

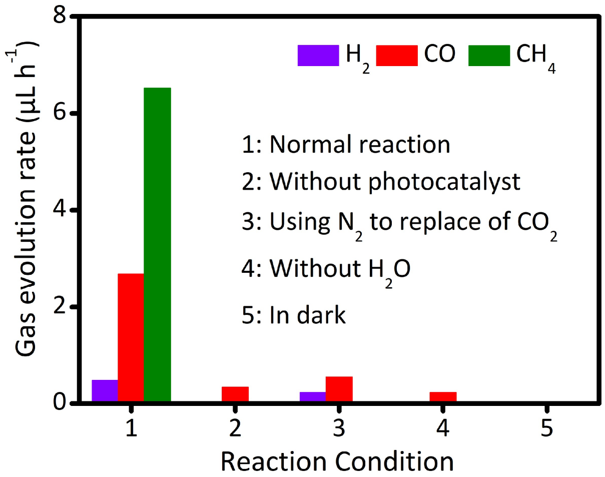

Supplementary Fig. 13 The $CuInSnS_4$ sample photoreduced CO_2 activity under different test conditions.

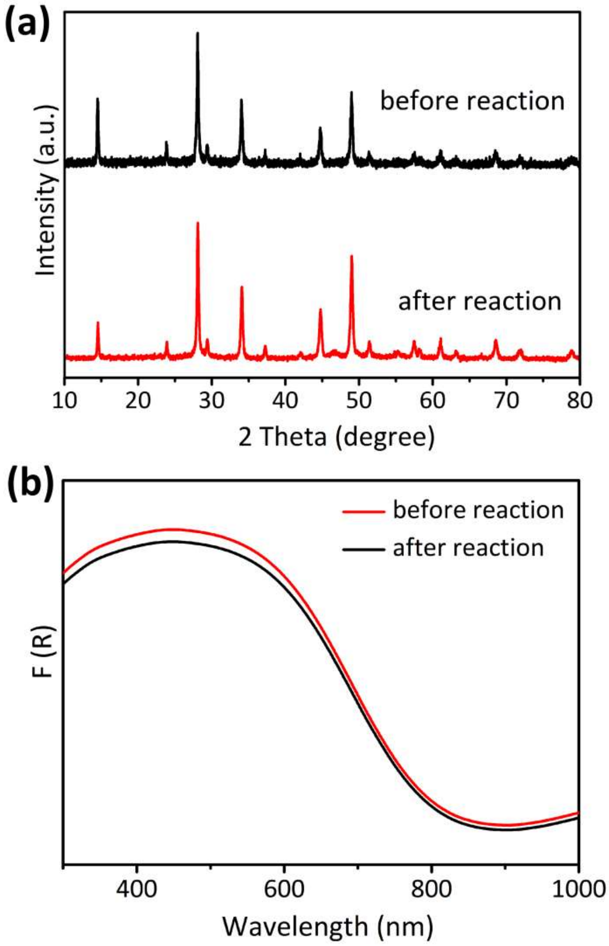

Supplementary Fig. 14 (a) XRD and (b) UV-Vis DRS of sample before and after the reaction.

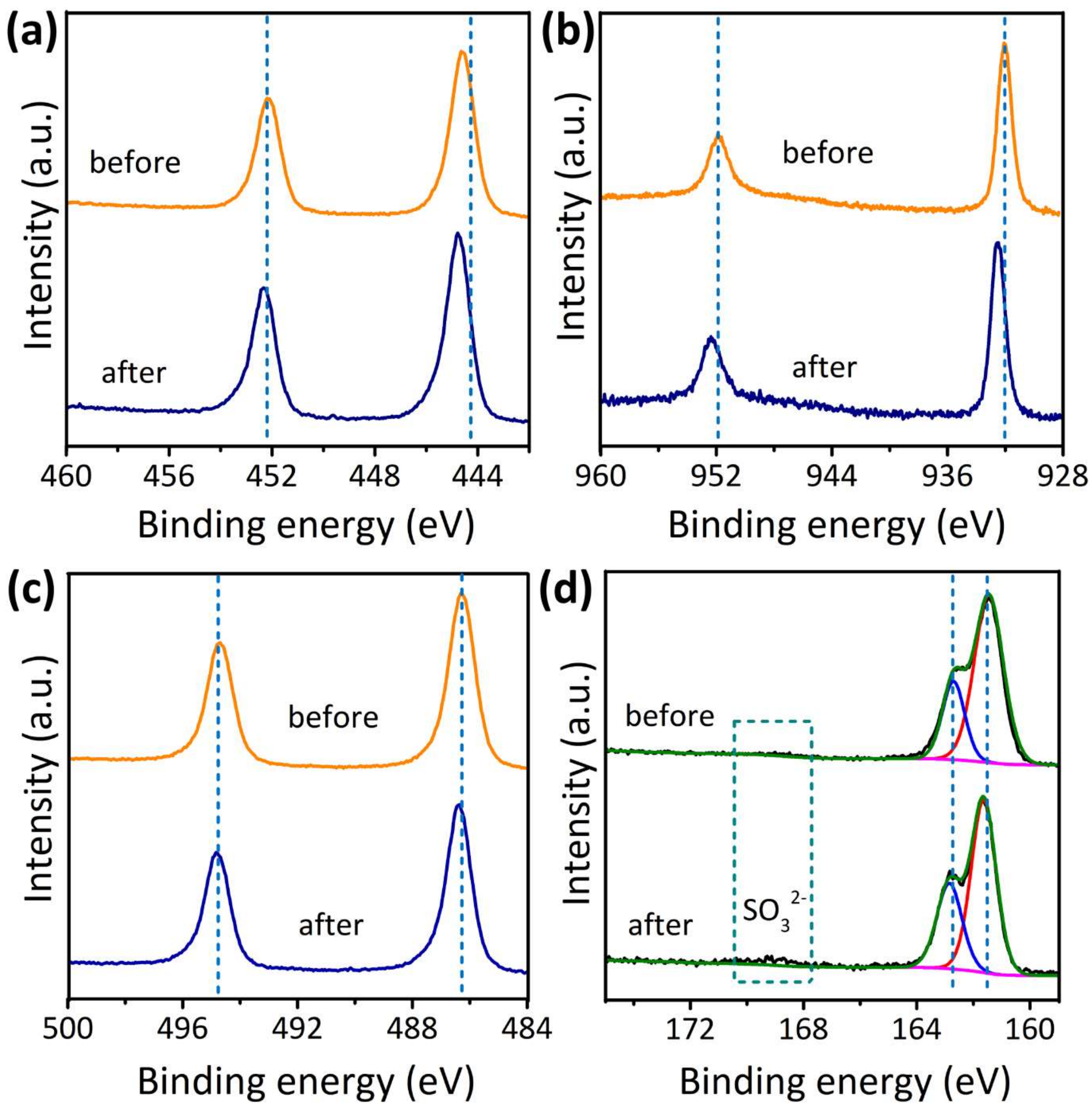

Supplementary Fig. 15 XPS spectra of $CuInSnS_4$ (180°C) before and after the reaction. (a) In*3d*, (b) Cu*2p*, (c) Sn*3d*, and (d) S*2p*.

The XPS spectra of the $CuInSnS_4$ photocatalyst before and after the reaction are presented in Supplementary Fig. 15. After the reaction, there is a slight increase in the binding energies of In, Cu, and S in the samples, while the binding energy of Sn remains relatively unchanged. Specifically, after the reaction, the binding energies of In$3d_{5/2}$ and In$3d_{3/2}$ in the $CuInSnS_4$ photocatalyst were measured to be 444.78 and 452.33 eV, respectively, indicating the presence of In^{3+} (Supplementary Fig. 15a). The Cu$2p_{3/2}$ and Cu$2p_{1/2}$ binding energies of

$CuInSnS_4$ sample, measured after the reaction, were measured to be 932.52 and 952.43 eV, respectively. This demonstrates that the valence state of Cu is +1 in the $CuInSnS_4$ sample (Supplementary Fig. 15b). Similarly, in the reacted $CuInSnS_4$ sample, the Sn$3d_{5/2}$ and Sn$3d_{3/2}$ doublets were centered at 486.42 and 494.84 eV, respectively, confirming the persistence of the Sn^{4+} valence state (Supplementary Fig. 15c). The binding energies of S$2p_{3/2}$ and S$2p_{1/2}$ in the $CuInSnS_4$ sample, measured after the reaction, were determined to be 161.66 and 162.84 eV, respectively, corresponding to the S^{2-} valence state (Supplementary Fig. 15d). Additionally, a stable S-C-O-In adsorption configuration is created on the (1 1 1) crystal plane of the $CuInSnS_4$ sample due to the adsorption and activation of CO_2 molecules. This adsorption configuration enables the transfer of electrons from the (1 1 1) facet of the $CuInSnS_4$ sample to CO_2 molecules, leading to the activation of CO_2 molecules. As a result, the charge density of the indium atoms decreases, and the binding energy increases. S atoms transfer electrons to carbon atoms of CO_2, while Cu atoms can transfer electrons to sulfur atoms. Therefore, the degree of increase in the binding energy of S atoms is lower than that of Cu atoms. It is worth noting that the surface lattice S^{2-} in the metal sulfide of the photocatalyst can be oxidized to SO_3^{2-} or SO_4^{2-} by photogenerated holes if the photocatalyst undergoes photocorrosion, resulting in changes in the surface structures of the metal sulfide in the composite catalysts[2,3]. However, the XPS spectrum of S element after the reaction shows only a doublet attributed to lattice S^{2-} and a very faint XPS peak of SO_3^{2-} species, as shown in Supplementary Fig. 15d. For the reacted catalyst, the XPS peaks with binding energies of 161.66 and 162.84 eV are assigned to S$2p_{3/2}$ and S$2p_{1/2}$, while the new peaks with binding energies in the range of 168.26~170.26 eV are assigned to the XPS peaks of SO_3^{2-} species[4,5]. The appearance of

SO_3^{2-} species indicates that photocorrosion occurs in the photocatalyst during the long-term reaction process.

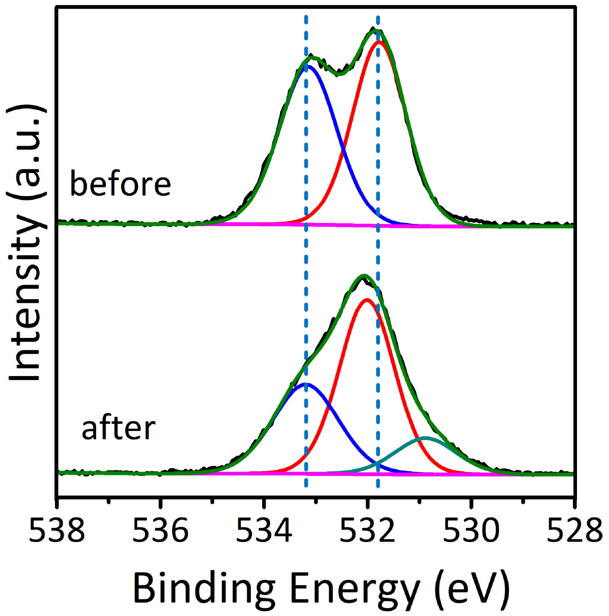

Supplementary Fig. 16 O*1s* spectra of $CuInSnS_4$ (180°C) photocatalyst before and after reaction.

Supplementary Fig. 16 shows the O*1s* spectra of the $CuInSnS_4$ photocatalyst before and after the reaction. For the catalyst before the reaction, a set of peaks with binding energies of 531.77 and 533.18 eV is assigned to surface hydroxyl groups (-OH) and surface adsorbed oxygen (O_2, CO_3^{2-}), respectively[6-8]. For the after reaction catalyst, a set of peaks with binding energies of 530.82, 532.09, and 533.20 eV is assigned to lattice oxygen, surface hydroxyl groups, and surface adsorbed oxygen, respectively. It is noted that after the reaction, the binding energy is 530.82 eV similar to lattice oxygen of In_2O_3 (530.76 eV)[9]. Compared to the sample before the reaction, the content of hydroxyl groups on the surface of the catalyst after the reaction remains basically unchanged, but the binding energy increases. Meanwhile, the adsorbed oxygen species on the surface of the catalyst decrease significantly after the

reaction. This may be due to the interaction between adsorbed oxygen species and photogenerated carriers that generates active oxygen species to further oxidize S^{2-} to SO_3^{2-}. Therefore, in the photocatalytic oxidation reaction, the photogenerated holes mainly oxidize the adsorbed oxygen and other species adsorbed on the catalyst surface to generate active species and then oxidize the catalyst surface S^{2-} to SO_3^{2-}.

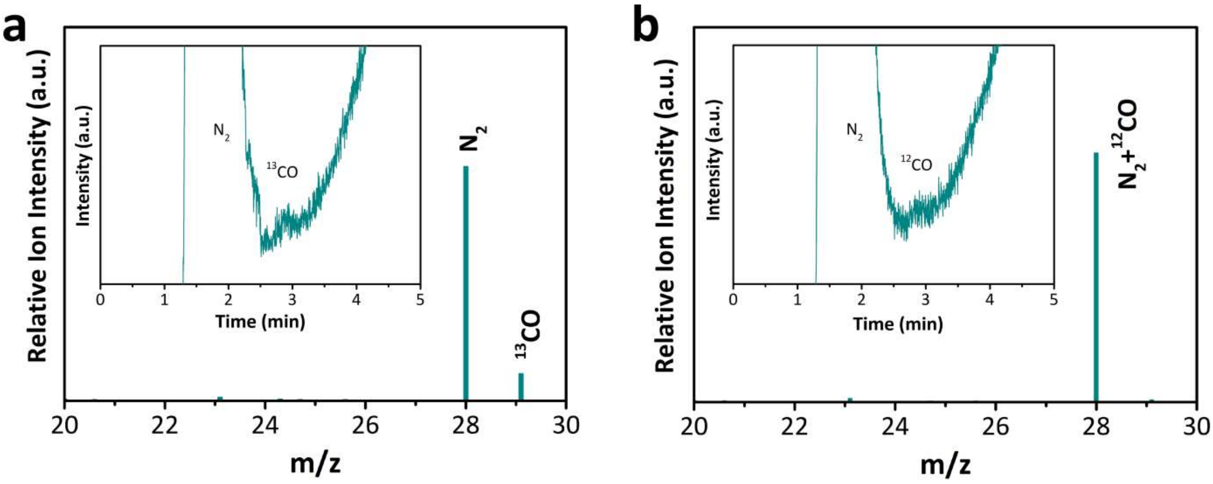

Supplementary Fig. 17 (a) GC-MS spectra of ^{13}CO generated from $^{13}CO_2$. (b) GC-MS spectra of ^{12}CO generated from $^{12}CO_2$.

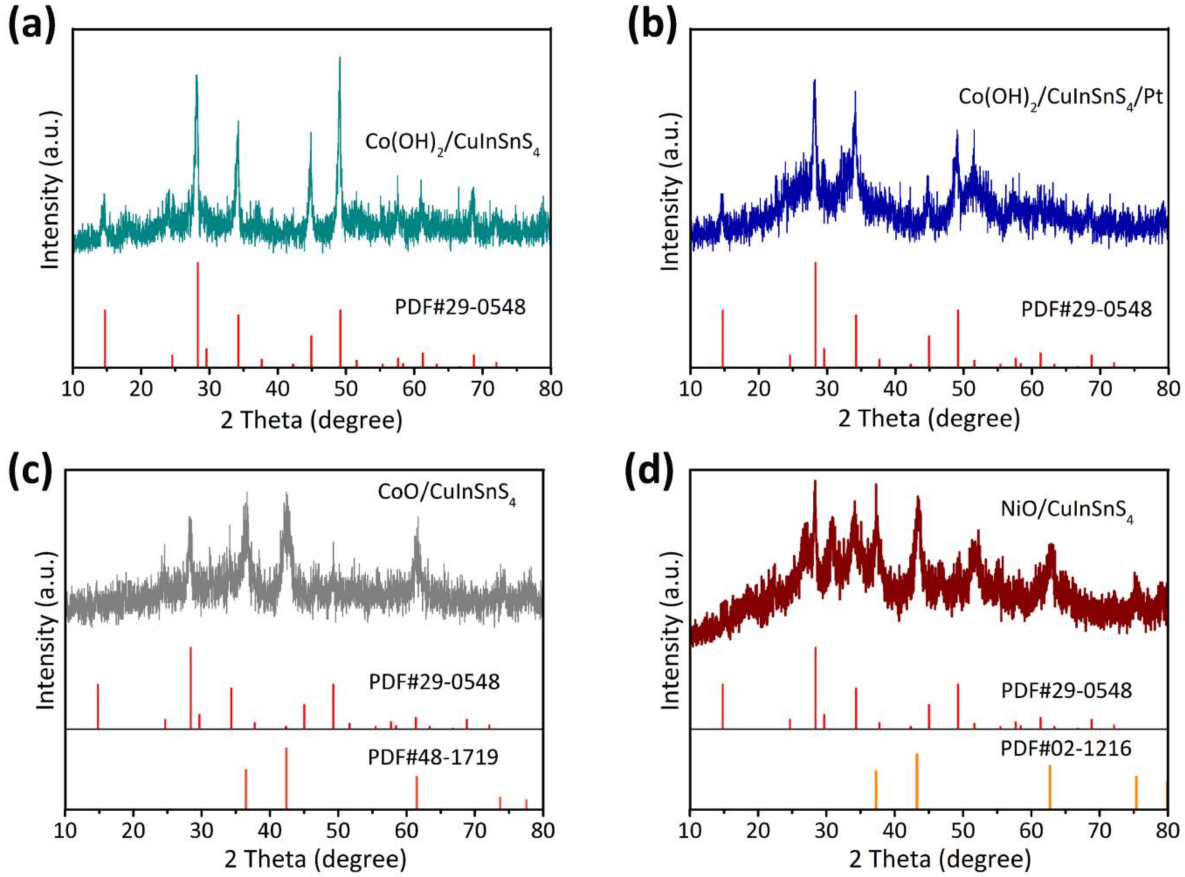

Supplementary Fig. 18 XRD patterns of (a) 5%$Co(OH)_2/CuInSnS_4$, (b) 5%$Co(OH)_2/CuInSnS_4$/1%Pt, (c) 10%$CoO/CuInSnS_4$, and (d) 10%$NiO/CuInSnS_4$.

The XRD patterns determined the crystal phase of $CuInSnS_4$ samples modified with a series of co-catalysts, as presented in Supplementary Fig. 18. In the case of 5%$Co(OH)_2$-modified $CuInSnS_4$ photocatalysts, as well as 5%$Co(OH)_2$ and 1%Pt co-modified $CuInSnS_4$ sample, the XRD patterns exhibited only the diffraction peaks corresponding to cubic $CuInSnS_4$. No distinct diffraction peaks for $Co(OH)_2$ or Pt species were observed. The absence of the diffraction peaks for Pt species could possibly be attributed to the low concentration. Additionally, the lack of clear diffraction peaks for $Co(OH)_2$ might arise from its relatively low crystallinity. In contrast, for NiO and CoO cocatalyst-modified $CuInSnS_4$ photocatalysts, the XRD analysis successfully detected diffraction peaks attributed to CoO and NiO. Both CoO and NiO co-catalysts exhibited patterns consistent with cubic CoO (PDF#48-1719) and NiO

(PDF#02-1216).

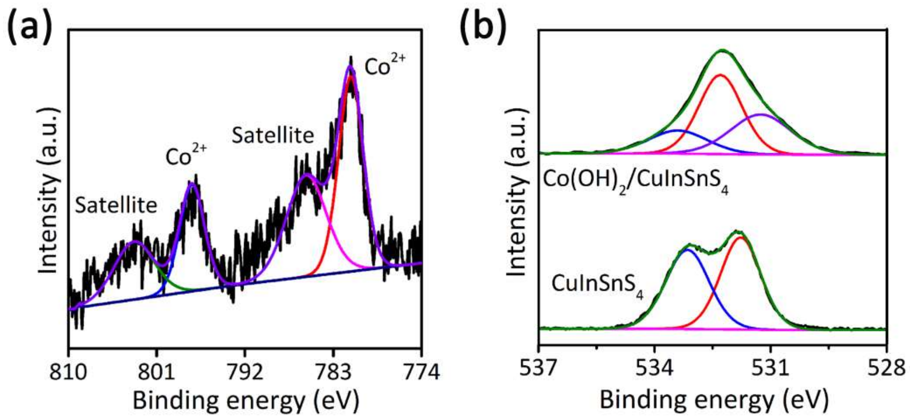

Supplementary Fig. 19 (a) Co*2p* and (b) O*1s* XPS spectra of 5%$Co(OH)_2/CuInSnS_4$ sample.

XPS was employed to confirm the chemical states of $Co(OH)_2$ cocatalyst. Supplementary Figure 19a illustrated Co*2p* XPS spectra in 5%$Co(OH)_2/CuInSnS_4$ sample. A set of peaks with binding energies of 781.27 and 797.42 eV corresponded to $Co2p_{3/2}$ and $Co2p_{1/2}$ of $Co(OH)_2$, respectively. It was noteworthy that the binding energy difference (Δ = 16.1 eV) between these two peaks proved the presence of Co in the form of $Co(OH)_2$, in accordance with findings from literature reports[10,11]. Furthermore, an additional set of peaks with binding energies of 785.72 and 803.29 eV represented the satellite peaks of Co^{2+} of $Co(OH)_2$ cocatalyst[12]. Additionally, O*1s* XPS spectra also revealed three peaks with binding energies of 531.25, 532.29, and 533.39 eV, which were ascribed to lattice oxygen of $Co(OH)_2$, absorbed hydroxyl, and surface absorbed H_2O or O_2, respectively[11,6,8] (Supplementary Fig. 19b). Therefore, $Co(OH)_2$ co-catalyst was successfully modified on the surface of $CuInSnS_4$ photocatalyst.

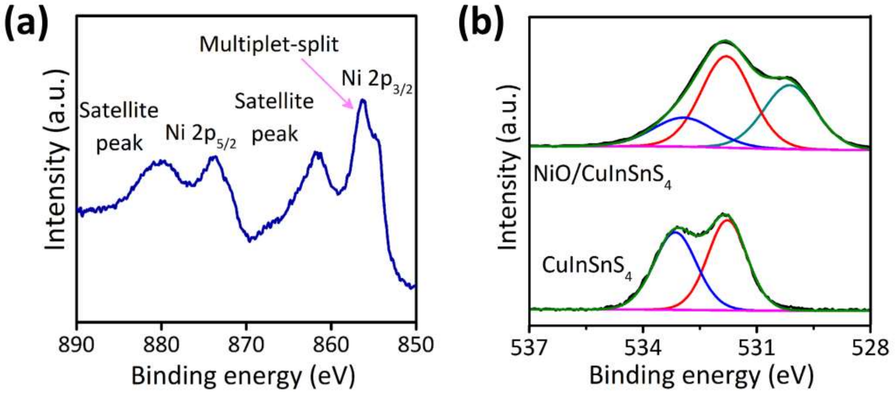

Supplementary Fig. 20 (a) Ni2*p* and (b) O1*s* XPS spectra of 10%NiO/$CuInSnS_4$ sample.

The chemical states of NiO cocatalyst was analyzed and confirmed by XPS. Supplementary Fig. 20a showed XPS spectra of the Ni2*p* in NiO/$CuInSnS_4$ sample. A group of peaks with binding energies of 855.50 and 873.68 eV corresponded to Ni$2p_{3/2}$ and Ni$2p_{1/2}$ of Ni^{2+}, respectively[13,14]. Another group of peaks with binding energies of 861.81 and 880.35 eV represented the satellite peaks of Ni^{2+} [15]. An obvious multiple splitting phenomenon in the main XPS peak of Ni$2p_{3/2}$ was consistent with NiO. Hence, it can be inferred that NiO was successfully supported on the surface of $CuInSnS_4$ sample. Supplementary Fig. 20b of O1*s* XPS spectra showed that the three peaks with binding energies of 530.15, 531.83, and 533.01 eV were assigned to lattice oxygen of NiO, surface hydroxyl, and surface adsorbed oxygen, respectively[6,8]. Notably, for pure $CuInSnS_4$, only the XPS peaks of surface hydroxyl groups and surface adsorbed oxygen at binding energies of 531.77 and 533.15 eV were detected. Hence, the XPS peak at 530.15 eV was assigned to the lattice oxygen of NiO[13].

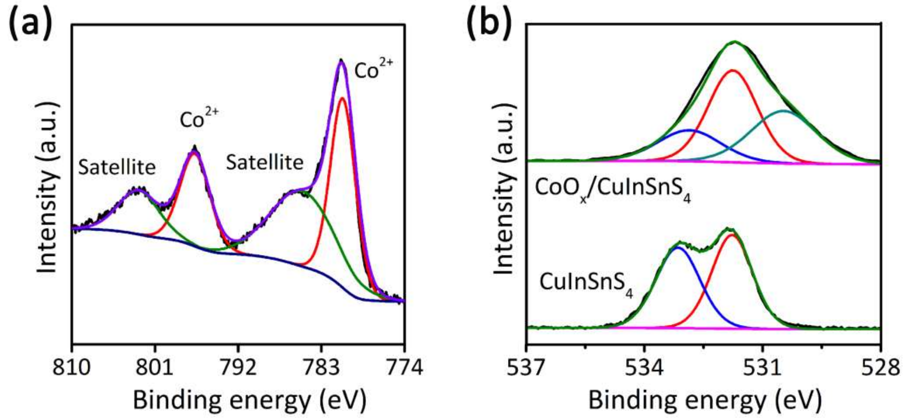

Supplementary Fig. 21 (a) Co*2p* and (b) O*1s* XPS spectra of 10%CoO/$CuInSnS_4$ sample.

The chemical states of the CoO cocatalyst were analyzed and verified using XPS. Supplementary Fig. 21a presented the XPS spectra of Co*2p* in the CoO/$CuInSnS_4$ sample. The Co*2p* XPS peaks at 780.76 eV (Co$2p_{3/2}$) and 796.80 eV (Co$2p_{1/2}$) revealed distinct characteristic peaks of the Co^{2+} oxidation state[16]. Moreover, their satellite peaks appeared at binding energies of 785.25 and 802.89 eV further confirming the presence of CoO. Supplementary Fig. 21b of O*1s* XPS spectra showed that the three peaks with binding energies of 530.53, 531.76, and 532.91 eV were assigned to lattice oxygen of CoO, surface hydroxyl, and surface adsorbed oxygen, respectively[6,8].

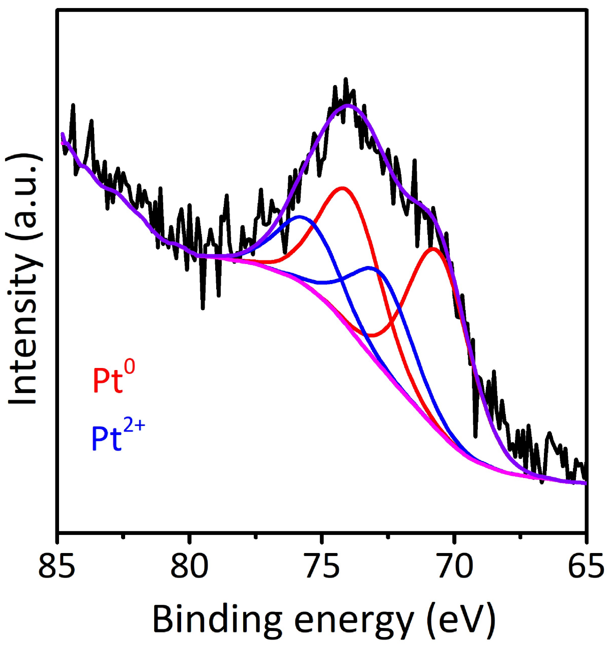

Supplementary Fig. 22 Pt*4f* XPS spectra of 1%Pt/$CuInSnS_4$ sample.

Supplementary Fig. 22 showed Pt*4f* XPS spectra of 1%Pt/$CuInSnS_4$ sample. A set of peaks with binding energies of 70.59 and 73.92 eV was assigned to Pt$4f_{7/2}$ and Pt$4f_{5/2}$ of Pt^0, respectively, while another set of peaks with binding energies of 72.72 and 75.50 eV was attributed to Pt$4f_{7/2}$ and Pt$4f_{5/2}$ of Pt^{2+}, respectively[17]. Evidently, Pt primarily existed on the surface of $CuInSnS_4$ sample in the form of metal Pt^0, accompanied by a part of PtO.

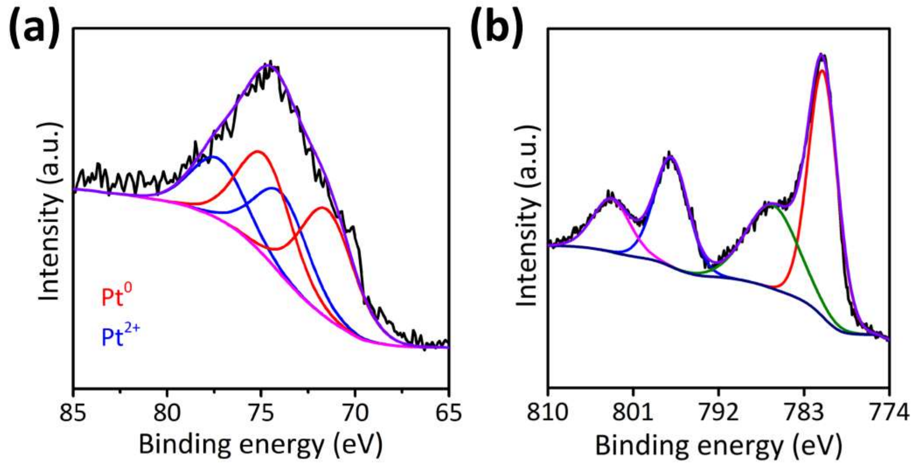

Supplementary Fig. 23 (a) Pt*4f* and (b) Co*2p* XPS spectra of 5%$Co(OH)_2$/$CuInSnS_4$/1%Pt sample.

XPS was employed for the chemical state of $Co(OH)_2$ and Pt cocatalysts in 5%$Co(OH)_2$/$CuInSnS_4$/1%Pt sample, as shown in the Supplementary Fig. 23. The two characteristic peaks with binding energies of 70.77 and 74.19 eV were assigned to Pt$4f_{7/2}$ and Pt$4f_{5/2}$ of Pt^0, respectively, while the two characteristic peaks with binding energies of 72.98 and 75.80 eV were assigned to Pt$4f_{7/2}$ and Pt$4f_{5/2}$ of Pt^{2+}, respectively[17]. As depicted in Supplementary Fig. 23b, the Co*2p* spectrum exhibited two characteristic peaks with binding energies of 781.13 and 797.15 eV, corresponding to Co$2p_{3/2}$ and Co$2p_{1/2}$[10,11]. The position and distance (Δ=16.02 eV) between these two peaks confirmed the existence of Co in the form of $Co(OH)_2$. The two characteristic peaks with binding energies of 786.49 and 803.56 eV represented the characteristic satellite peaks of Co^{2+} [12]. Therefore, it can be determined that the $Co(OH)_2$ and Pt cocatalysts were successfully deposited on the surface of $CuInSnS_4$ sample.

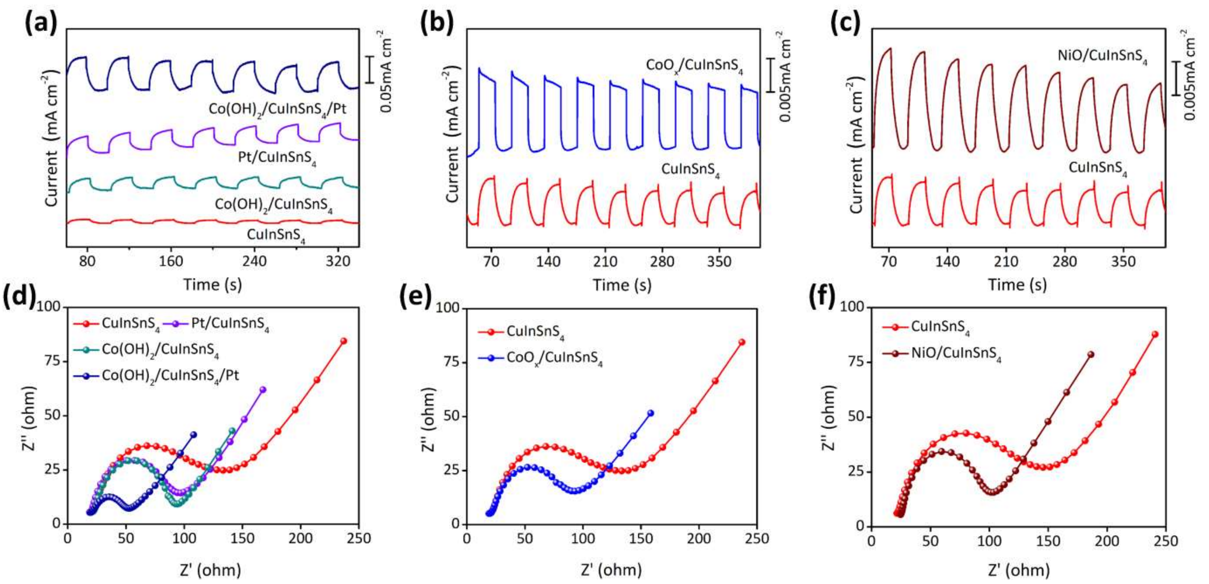

Supplementary Fig. 24 Photocurrent response and electrochemical impedance spectroscopy of the as-prepared samples.

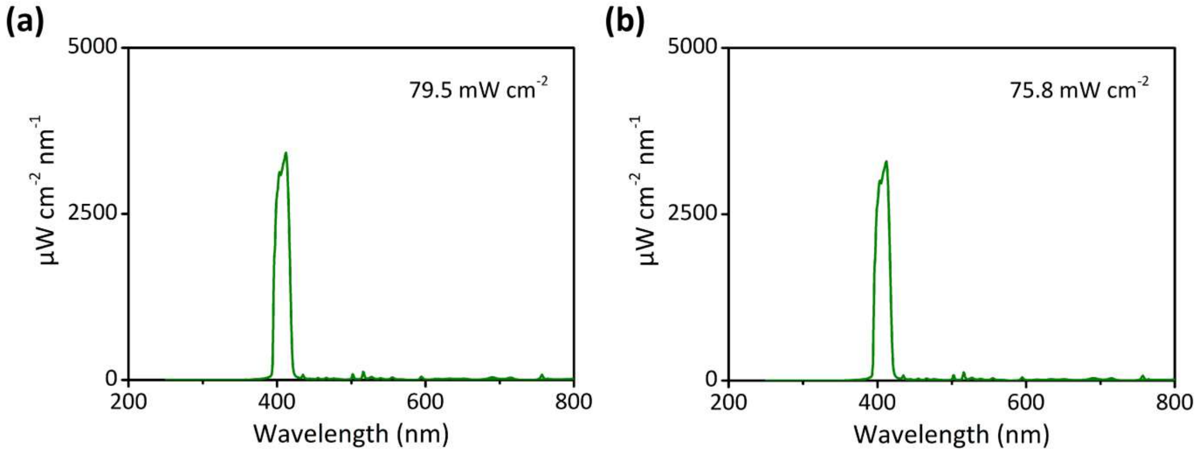

Supplementary Fig. 25 Spectrum and intensity of 400 nm monochromatic light. (a) First test, and (b) Second test.

The spectrum and light intensity of 400 nm monochromatic light were measured twice in parallel, as shown in Supplementary Fig. 25. The light intensity values for the two measurements were 79.5 mW cm^{-2} and 75.8 mW cm^{-2}, respectively. Therefore, the average value of the two measured values was taken as the light intensity value of the 400 nm monochromatic light. The calculation formula for apparent quantum efficiency was as

followings.

$$N_{photos} = \frac{I \times S \times T}{h\frac{c}{\lambda}} = \frac{77.65 \times 1.4 \times 1.4 \times 3.14 \times 10^{-3} \times 3600}{6.626 \times 10^{-34} \times \frac{3 \times 10^{8}}{400 \times 10^{-9}}} = 3.46 \times 10^{21}$$

$$N_{CH_4} = 1.165 \times 10^{-6} \times 6.02 \times 10^{23} = 7.01 \times 10^{17}$$

$$N_{CO} = 0.375 \times 10^{-6} \times 6.02 \times 10^{23} = 2.23 \times 10^{17}$$

$$400\text{nm} \quad AQY_{CH_4} = \frac{N_{CH_4} \times 8}{N_{photos}} = \frac{7.01 \times 10^{17} \times 8}{3.46 \times 10^{21}} \times 100\% = 0.16\%$$

$$400\text{nm} \quad AQY_{CO} = \frac{N_{CO} \times 2}{N_{photos}} = \frac{2.23 \times 10^{17} \times 2}{3.46 \times 10^{21}} \times 100\% = 0.01\%$$

I: Light intensity (mW cm^{-2}), S: Catalyst illumination area (cm^{-2}), T: illumination Time (h), h: Planck's constant, c: Speed of light, λ: Wavelength.

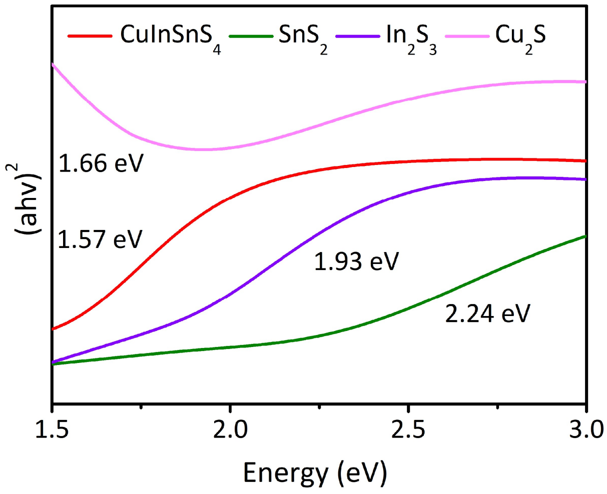

Supplementary Fig. 26 The optical band gap energy (E_g) of the corresponding $CuInSnS_4$ and various single metal sulfides.

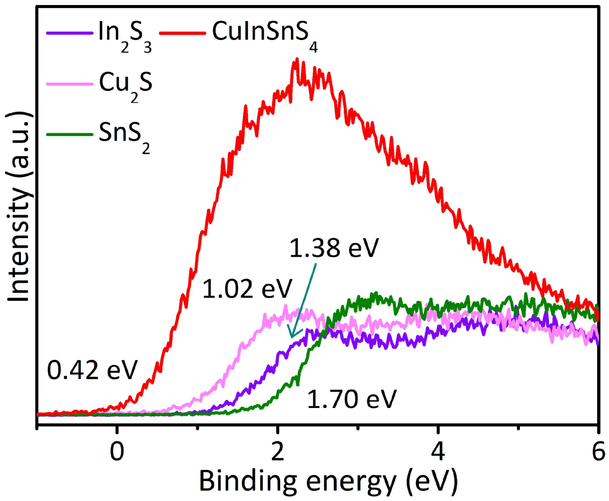

Supplementary Fig. 27 Valence-band XPS spectra of the $CuInSnS_4$ and various single metal sulfides.

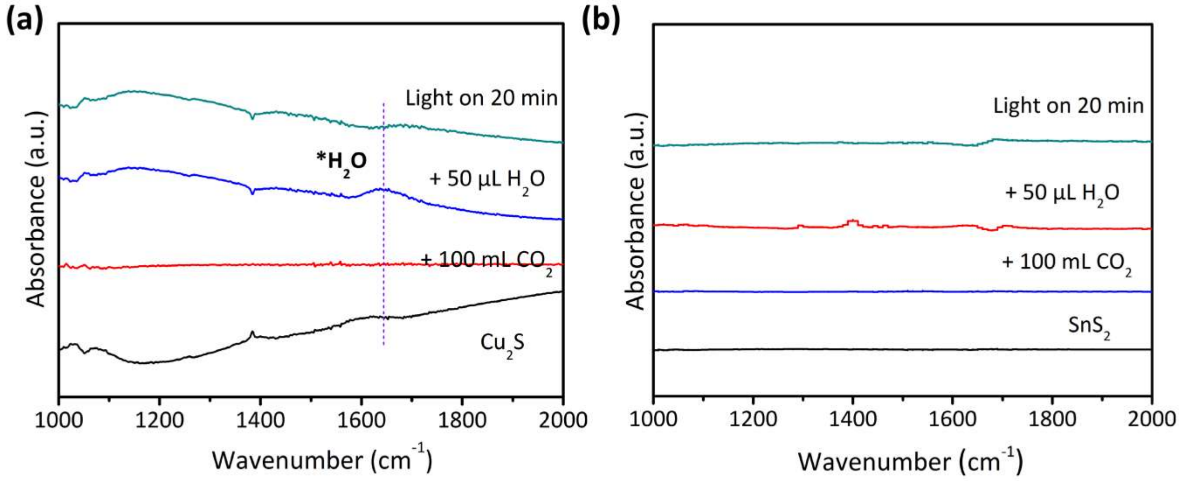

Supplementary Fig. 28 In situ FT-IR spectra of CO_2 adsorbed on different photocatalysts. (a) Cu_2S, (b) SnS_2. All the spectra are the difference spectra between after and before CO_2 adsorption.

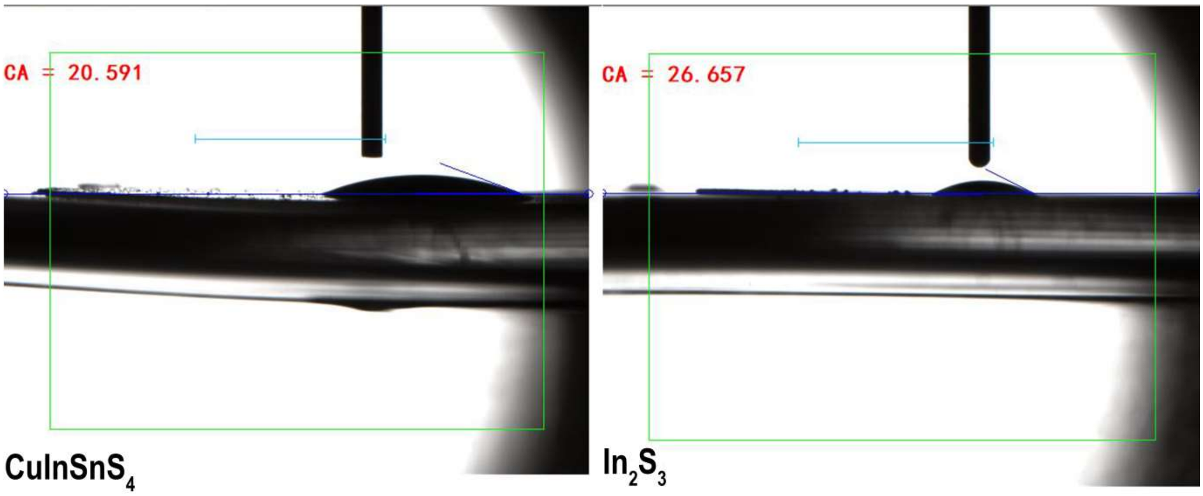

Supplementary Fig. 29 Contact angles of $CuInSnS_4$ and In_2S_3.

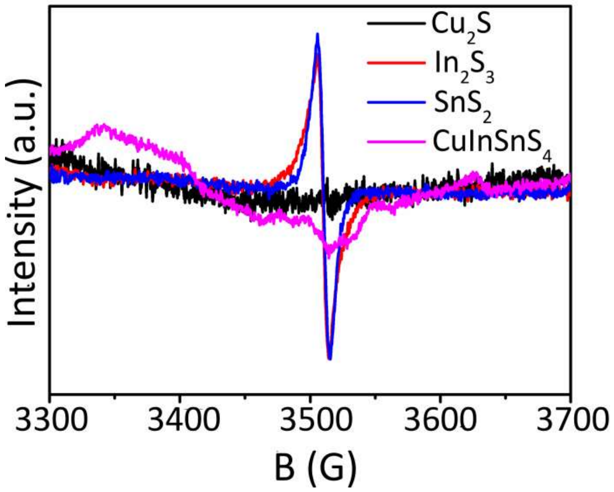

Supplementary Fig. 30 EPR spectra of various metal sulfides.

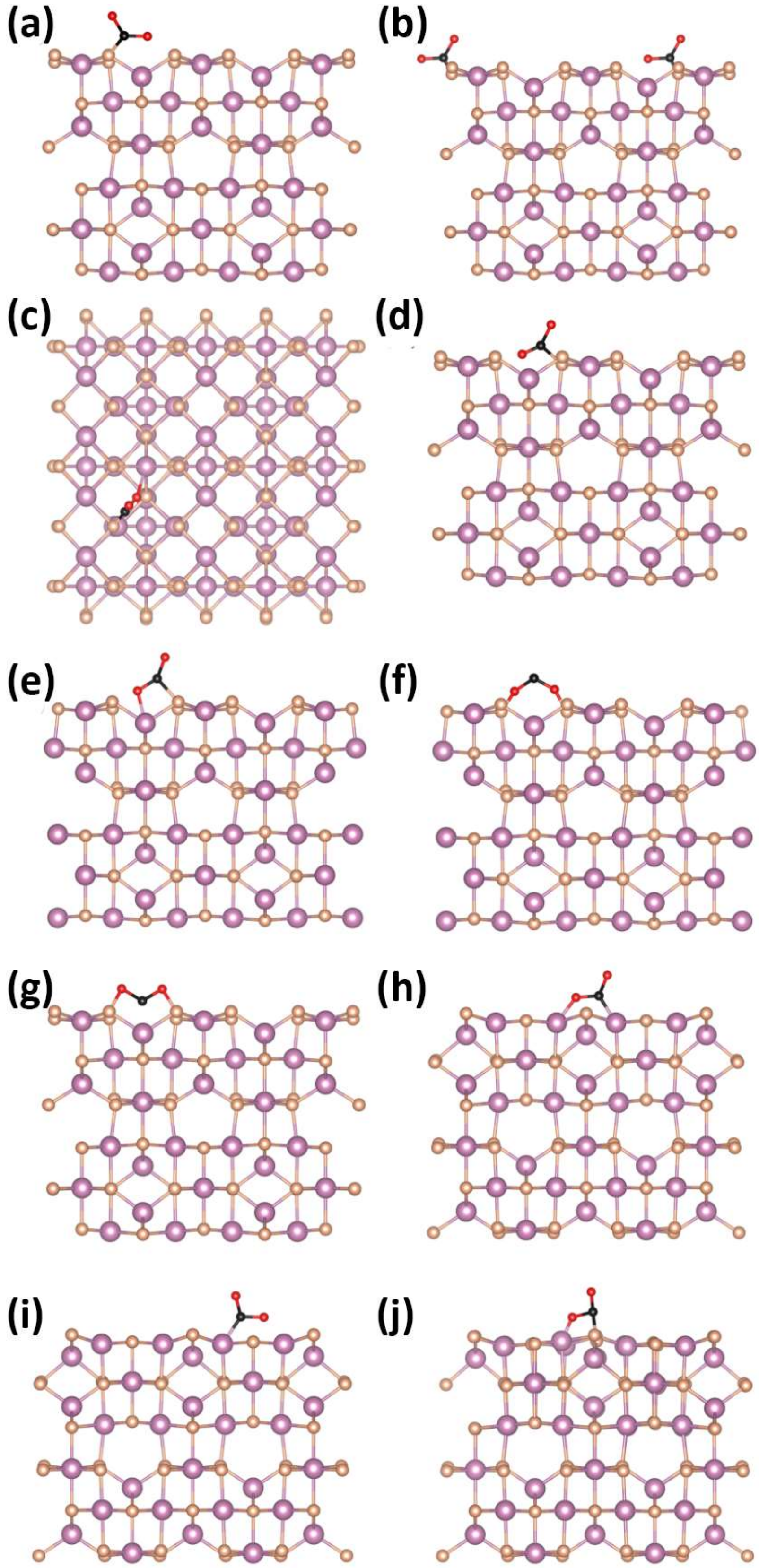

Supplementary Fig. 31 Theoretically designed adsorption configuration of CO_2 on the In_2S_3 surface.

The different adsorption configurations of CO_2 on the In_2S_3 photocatalyst surface are designed and established, as shown in Supplementary Fig. 31. However, after quantum chemistry calculation and optimization processing, it is found that the above-mentioned adsorption configurations cannot achieve effective adsorption of CO_2 molecules. Therefore, the above-mentioned adsorption configurations are excluded as an effective adsorption configuration for CO_2 on the surface of the In_2S_3 photocatalyst.

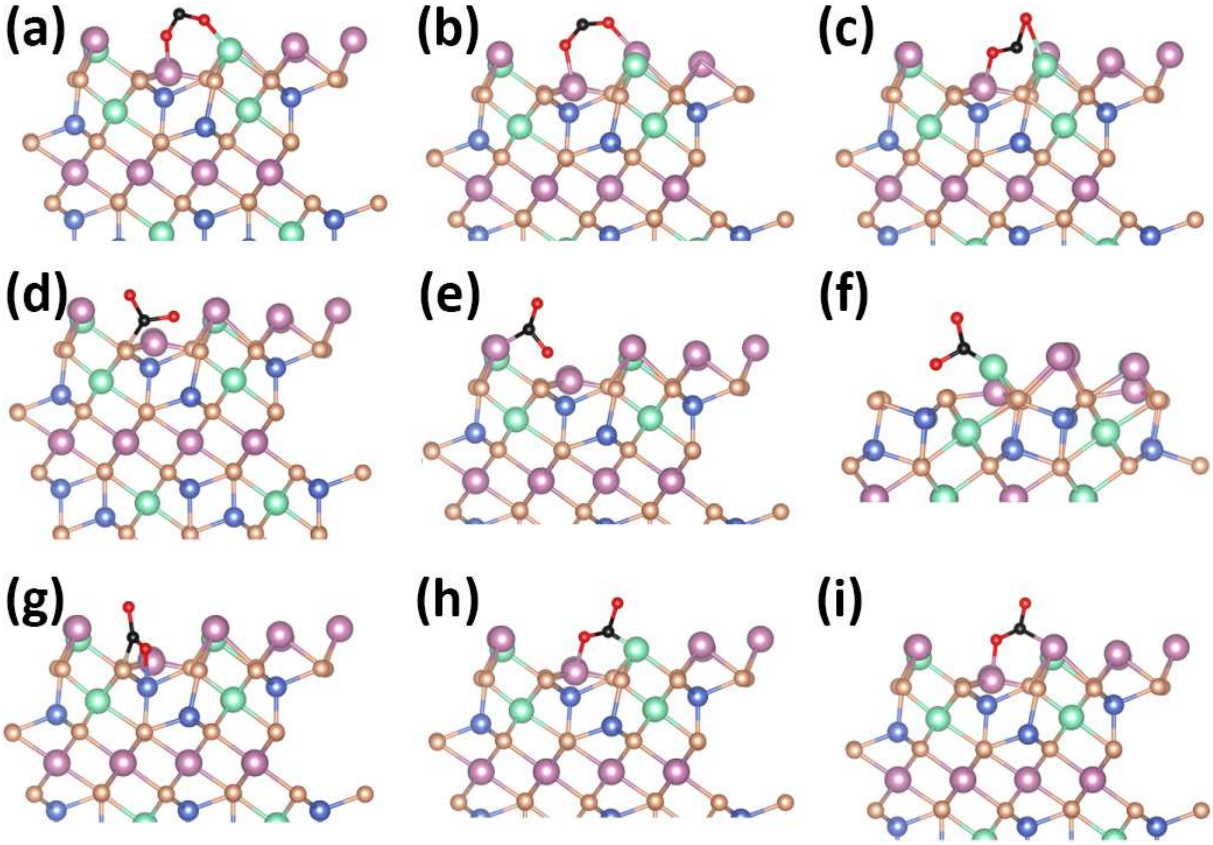

Supplementary Fig. 32 Theoretically designed adsorption configuration of CO_2 on the $CuInSnS_4$ surface.

The different adsorption configurations of CO_2 on the $CuInSnS_4$ photocatalyst surface are established, as shown in Supplementary Fig. 32. Obviously, the structural models of different metal atoms as CO_2 adsorption sites were established respectively. However, after quantum

chemistry calculation and optimization processing, it is found that all the above adsorption configurations are invalid adsorption configurations. Moreover, In our study, based on the in-situ infrared spectra analysis, we observed the binding of C with S rather than In, Cu or Sn. When the C atom of CO_2 binds to the S atom on the (1 1 1) crystal plane of the $CuInSnS_4$ sample, the O atom can only form a bond with the In atom and not with the Cu or Sn atoms. This is due to the distribution of atoms on the (1 1 1) crystal plane of the $CuInSnS_4$ sample. The (1 1 1) crystal plane primarily consists of S atoms, In atoms, and some Sn atoms, while the Cu atoms are predominantly located in the deeper layers, as depicted in the figure. Consequently, when the C atom of the CO_2 molecule bonds with the S atom on the (1 1 1) crystal plane, the O atom cannot form a bond with the Cu atom situated deeper within the crystal plane. Simultaneously, we provide a computational model for Cu atoms as potential CO_2 adsorption sites, as depicted in Supplementary Fig. 32g. However, upon optimization, it was discovered that the aforementioned adsorption models were all deemed invalid configurations. Consequently, Cu atoms were excluded as reactive sites for CO_2 adsorption activation. Furthermore, we also examined the bonded structures of O atoms and Sn atoms individually, as shown in Supplementary Fig. 32a and Supplementary Fig. 32c. Our findings indicate that the adsorption configuration involving O atoms bonded to Sn atoms is ineffective compared to O atoms bonded to In atoms. In other words, when C atoms are adsorbed onto S atoms and O atoms are adsorbed onto Sn atoms, this particular adsorption configuration is unstable. Hence, we have excluded the possibility of the O atom in the CO_2 molecule bonding to the Sn atom. Therefore, the above-mentioned adsorption configurations are excluded as an effective adsorption configuration for CO_2 on the surface of the $CuInSnS_4$ photocatalyst.

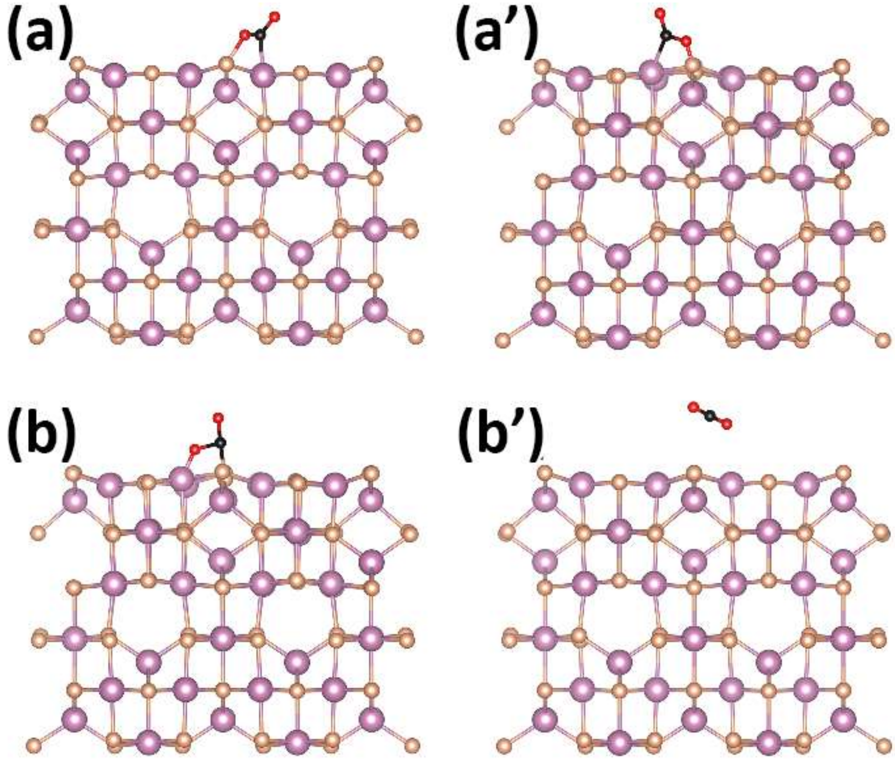

Supplementary Fig. 33 Theoretically designed adsorption configuration of CO_2 molecules on the In_2S_3 surface and the corresponding adsorption configuration after optimization. (a) Theoretically designed S-O-C-In adsorption configuration. (a') The optimized S-O-C-In adsorption configuration. (b) Theoretically designed In-O-C-S adsorption configuration. (b') The optimized In-O-C-S adsorption configuration.

Supplementary Fig. 33a shows the theoretically designed adsorption configuration of CO_2 on the In_2S_3 photocatalyst surface. It can be clearly seen that the adsorption configuration is S-O-C-In. Fortunately, the adsorption configuration can still exist stably after optimization, as shown in Supplementary Fig. 33a'. On the contrary, if the C atom in the CO_2 molecule bond with the S atom on the In_2S_3 surface, the O atom bond with the In atom to form an In-O-C-S adsorption configuration, as shown in Supplementary Fig. 33b. However, after optimization, it is found that the adsorption configuration is an invalid adsorption configuration, as shown in Supplementary Fig. 33b'. Therefore, Supplementary Fig. 33a' is chosen as the stable adsorption configuration of CO_2 on the surface of In_2S_3.

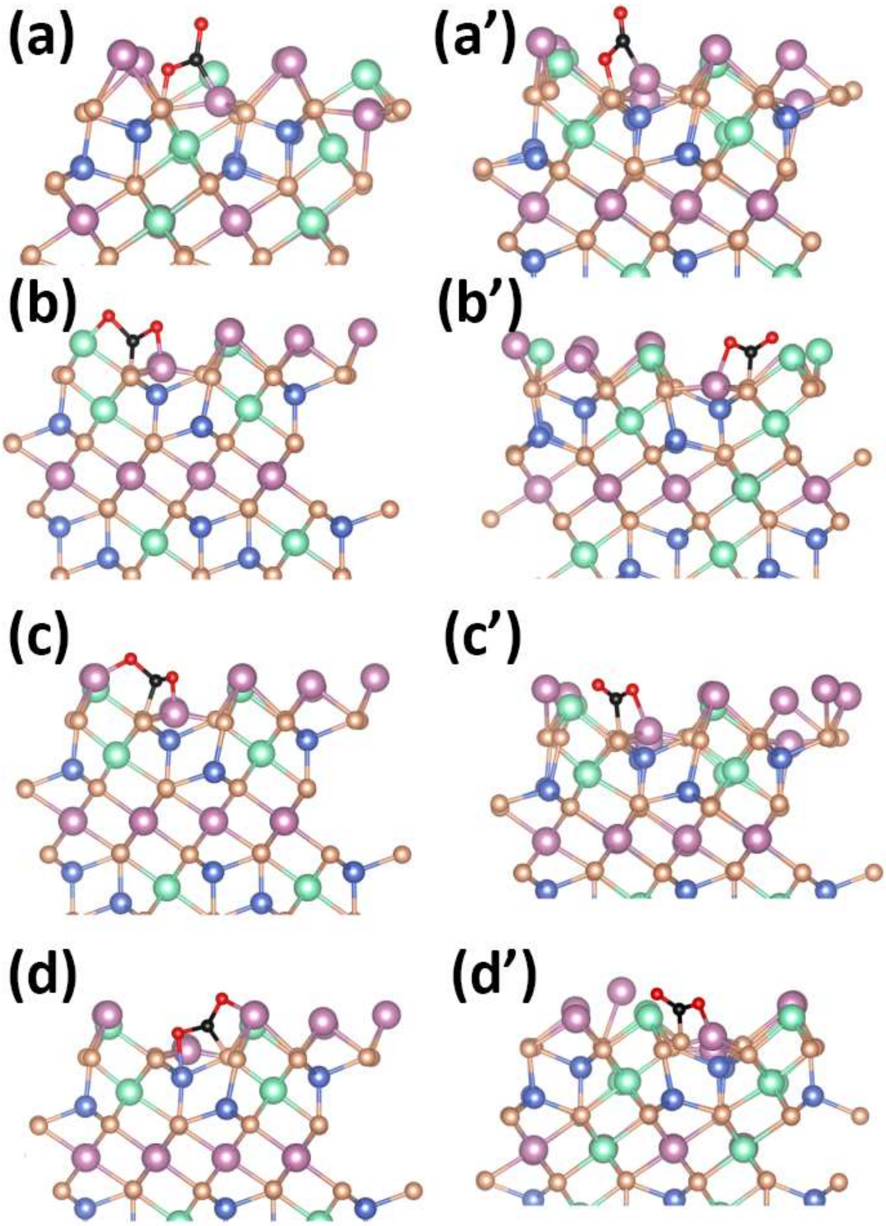

Supplementary Fig. 34 Theoretically designed adsorption configuration of CO_2 on the $CuInSnS_4$ surface.

The different adsorption configurations of CO_2 on the $CuInSnS_4$ photocatalyst surface are established respectively. The crystal plane of the $CuInSnS_4$ sample (1 1 1) is mainly composed of In atoms and S atoms, so CO_2 can only rent with In atoms and S atoms to form a stable adsorption configuration. Therefore, it cannot interact with Cu atoms or Sn atoms to form a stable adsorption configuration. After optimization, the four stable adsorption configurations can be obtained as shown in Supplementary Fig. 34a'–Supplementary Fig. 34d'. The 4

adsorption configurations are divided into 2 categories, namely S-O-C-In and S-C-O-In adsorption configurations. Most importantly, the CO_2 adsorption energy in the S-C-O-In adsorption configuration is more negative than in the S-O-C-In adsorption configuration. In comparison, the CO_2 adsorption energy of the S-C-O-In adsorption configuration is about 1.63 eV lower than that of the S-O-C-In adsorption configuration. Therefore, S-C-O-In is selected as the stable adsorption configuration of CO_2 molecules on the $CuInSnS_4$ photocatalyst surface.

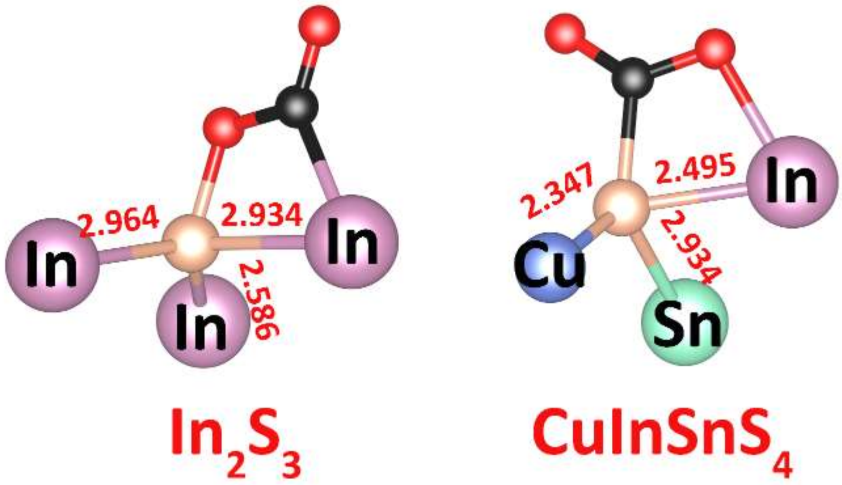

Supplementary Fig. 35 Coordination mode of S atom on the surface of (a) In_2S_3 and (b) $CuInSnS_4$.

The different coordination environments of S atom on the surface of In_2S_3 and $CuInSnS_4$ and the difference in charge density on the S atom lead to different adsorption configurations for CO_2, as shown in Supplementary Fig. 35 and Table S3. The S sulfur atom of the In_2S_3 (0 0 1) crystal plane mainly coordinates with 3 In atoms. In contrast, the S sulfur atom on the $CuInSnS_4$ (1 1 1) crystal plane mainly coordinates with 1 In atom, 1 Cu atom, and 1 Sn atom. Different coordination environments can directly optimize the electronic state of the S site, leading to coordinate bonding with the O and C atoms in the CO_2 molecule, respectively.

Before In_2S_3 and $CuInSnS_4$ form a stable CO_2 adsorption configuration, the surface S atom obtain electrons from the surrounding coordination metal atom and the charges are 6.240e and 6.158e, respectively. The different number of charges is because In_2S_3 and $CuInSnS_4$ each expose different crystal planes and transfer different amounts of charge between coordinating atom and S atom. Therefore, the S atom on the exposed (0 0 1) crystal plane of In_2S_3 is electron-rich sites, while S atom on $CuInSnS_4$ crystal (1 1 1) is electron-poor center. The electron-rich S sites on the exposed (0 0 1) crystal plane of In_2S_3 are bonded to O atom of CO_2 to a stable adsorption configuration. On the contrary, the electron-poor S center on $CuInSnS_4$ crystal (1 1 1) plane is more favorable to bond with with C atoms of CO_2 as a stable adsorption configuration.

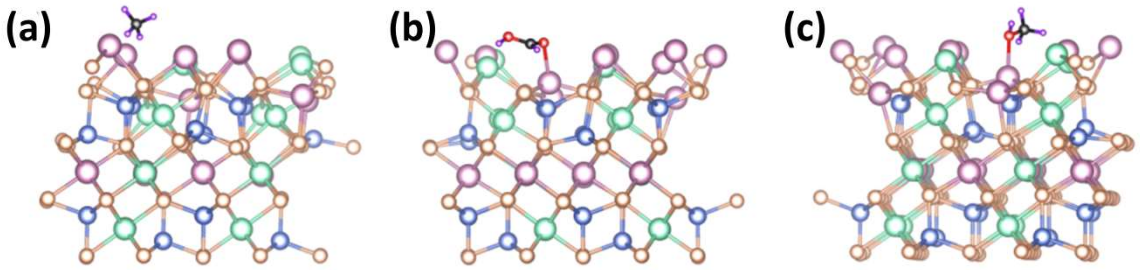

Supplementary Fig. 36 The adsorption model of CH_4, CH_3OH and HCOOH on the surface of $CuInSnS_4$ photocatalyst.

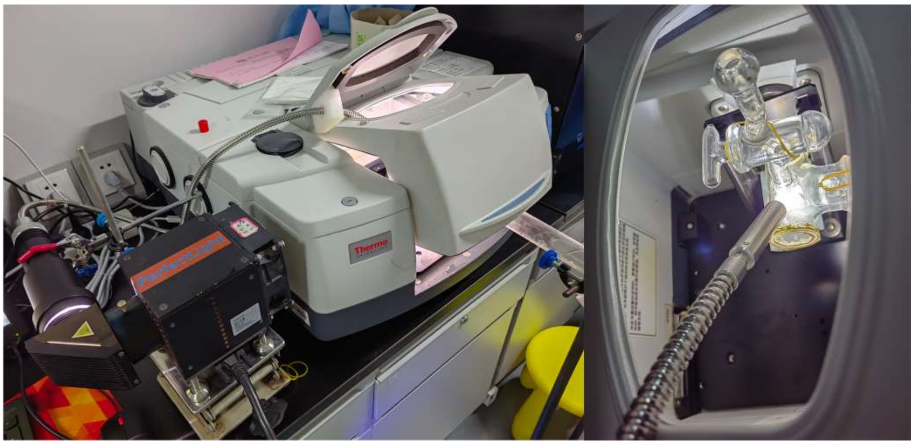

Supplementary Fig. 37 In situ infrared testing device.

Table S1 ICP-MS test of each element of $CuInSnS_4$ sample.

Sample	Quality m_0 (g)	Elements of the test	Sample element content (W)	Sample element molar (mmol)
		Cu	10.40%	0.0887
		Cu	10.46%	0.0892
		Cu	10.44%	0.0890
		Sn	17.40%	0.0794
$CuInSnS_4$	0.0542	Sn	17.66%	0.0806
		Sn	17.66%	0.0806
		In	16.91%	0.0798
		In	16.86%	0.0796
		In	16.96%	0.0801

Table S2 Comparing the photocatalytic CO_2 reduction performance of $CuInSnS_4$, modified $CuInSnS_4$, and common photocatalysts.

Catalyst	Generation rate of CH_4 (μmol h^{-1} g^{-1})	Generation rate of CO (μmol h^{-1} g^{-1})	Selectivity of CH_4	Ref.
$CuInSnS_4$	5.83	2.40	67.3%	This work
1%Pt/$CuInSnS_4$	43.25	7.85	84.6%	This work
10%CoO/$CuInSnS_4$	33.30	9.36	78.1%	This work
10%NiO/$CuInSnS_4$	11.80	6.85	63.3%	This work
3%$Co(OH)_2$/$CuInSnS_4$	18.5 0	3.02	86.0%	This work
5%$Co(OH)_2$/$CuInSnS_4$	145.45	32.32	81.8%	This work
8%$Co(OH)_2$/$CuInSnS_4$	46.80	13.22	78.0%	This work
10%$Co(OH)_2$/$CuInSnS_4$	18.30	6.20	74.7%	This work
5%$Co(OH)_2$/$CuInSnS_4$/1%Pt	195.60	22.00	89.9%	This work
Mn(II)-metalated porphyrin	~53	~21	71.2%	18
CuS@$ZnIn_2S_4$/C_{60}	43.6	6.4	87.2%	19
Pt@h-BN	9.24	0	100%	20
SnS_2/TiO_2	23	2.5	90.2%	21
SiC@MoS_2	14.42	0	100%	22
V_o-Zn-CoO	17.10	9.70	63.8%	23
In_4SnS_8	23.88	20.96	57.0%	24

V_S-$CuIn_5S_8$ single-unit-cell layers	8.70	0	100%	25
Cu_2O-111-Cu^0	78.40	0	97%	26
H-TiO_{2-x}(200)	16.20	4.2	79%	27
Ag_4/Cu_2O@rGO	82.6	4.0	95.4%	28
Cs_2SnI_6/SnS_2	~6.09	0	~100%	29
Pt-Cu_2O/TiO_2	1.42	0.05	96.6%	30
N-RGO/CdS	0.33	2.59	11.3%	31
$Au_{SA}/Cd_{1-x}S$	11.3	32.2	22.0%	32
Au_{SA}/CdS_{1-x}	0.40	3.70	9.3%	32
Ni-doped CoS_2 nanosheets	101.8	37.5	~73.10%	33
Pd_7Cu_1−TiO_2	19.60	1.90	95.9%	34
Ag_2Pd_1/TiO_2	79.0	0	100%	35

Table S3 Quantify the electron transfer between the surface and CO_2.

Element	Valence electron	In_2S_3 + CO_2	In_2S_3	Subtract	$CuInSnS_4$ + CO_2	$CuInSnS_4$	Subtract
In	13	12.688	12.661	+0.027	12.628	12.644	-0.036
S	6	6.022	6.240	-0.218	6.052	6.158	-0.106
C	4	3.958	3.700	+0.235	3.851	3.700	+0.151
O^1	6	6.129	6.150	-0.021	6.234	6.150	+0.084
O^2	6	6.220	6.150	+0.070	6.235	6.150	+0.085

Supplementary References

1. Momma, K., & Izumi, F. *VESTA 3* for three-dimensional visualization of crystal, volumetric and morphology data. *J. Appl. Crystallogr.* **44**, 1272–1276 (2011).

2. Weng, B., Qi, M. Y., Han, C., Tang, Z. R., & Xu, Y. J. Photocorrosion inhibition of semiconductor-based photocatalysts: basic principle, current development, and future perspective. *ACS Catal.* **9**, 4642–4687 (2019).

3. Yu, H., Huang, X., Wang, P., & Yu, J. Enhanced photoinduced stability and photocatalytic activity of CdS by dual amorphous cocatalysts: synergistic effect of Ti(IV)-hole cocatalyst and Ni(II)-electron cocatalyst. *J. Phys. Chem. C* **120**, 3722−3730 (2016).

4. Weide, P., Schulz, K., Kaluza, S., Rohe, M., Beranek, R., & Muhler, M. Controlling the photocorrosion of zinc sulfide nanoparticles in water by doping with chloride and cobalt ions. *Langmuir*, **32**, 12641−12649 (2016).

5. Ding, Y., Chen, Y., Guan, Z., Zhao, Y., Lin, J., Jiao, Y., & Tian, G. Hierarchical CuS@$ZnIn_2S_4$ hollow double-shelled p–n heterojunction octahedra decorated with fullerene C_{60} for remarkable selectivity and activity of CO_2 photoreduction into CH_4. *ACS Appl. Mater. Interfaces*, **14**, 7888–78992022 (2022).

6. Jiang, F., Wang, S., Liu, B., Liu, J., Wang, L., Xiao, Y., Xu, Y., & X. Liu. Insights into the Influence of CeO_2 Crystal Facet on CO_2 Hydrogenation to Methanol over Pd/CeO_2 Catalysts. *ACS Catal. ACS Catal.* **10**, 11493−11509 (2020).

7. Wang, N., Li, S., Zong, Y., & Yao, Q. Sintering inhibition of flame-made Pd/CeO_2 nanocatalyst for low-temperature methane combustion. *J. Aerosol Sci.* **105**, 64−72 (2017).

8. Wang, B., Chen, B., Sun, Y., Xiao, H., Xu, X., Fu, M., Wu, J., Chen, L., & Ye, D. Effects of dielectric barrier discharge plasma on the catalytic activity of Pt/CeO_2 catalysts. *Appl. Catal., B.* **238**, 328−338 (2018).

9. Sabri, M. M., Jung, J., Yoon, D. H., Yoon, S., Tak, Y. J., & Kim, H. J. Hydroxyl radical-assisted decomposition and oxidation in solution-processed indium oxide thin-film transistors. *J. Mater. Chem. C.* **3**, 7499-7505 (2015).

10. Yang, J., Liu, H., Martens, W. N., & Frost, R. L. Synthesis and characterization of cobalt hydroxide, cobalt oxyhydroxide, and cobalt oxide nanodiscs. *J. Phys. Chem. C* **114**, 111–119 (2010).

11. Luo, Y., Li, X., Cai, X., Zou, X., Kang, F., Cheng, H.-M., & Liu, B. Two-dimensional MoS_2 confined $Co(OH)_2$ electrocatalysts for hydrogen evolution in alkaline electrolytes. *ACS Nano* **12**, 4565–4573 (2018).

12. Pal, D., Sarkar, A., Ghosh, N. G., Sanke, D. M., Maity, D., Karmakar, K., Sarkar, D., Zade, S. S., & Khan, G. G. Integration of $LaCo(OH)_x$ photo-Electrocatalyst and plasmonic gold nanoparticles with Sb-doped TiO_2 nanorods for photoelectrochemical water oxidation. *ACS Appl. Nano Mater.* **4**, 6111–6123 (2021).

13. Zhang, P.-F., Zhang, J.-Y., Sheng, T., Lu, Y.-Q., Yin, Z.-W., Li, Y.-Y., Peng, X.-X., Zhou, Y., Li, J.-T., Wu, Y.-J., Lin, J.-X., Xu, B.-B., Qu, X.-M., Huang, L., & Sun, S.-G. Synergetic effect of Ru and NiO in the electrocatalytic decomposition of Li_2CO_3 to enhance the performance of a Li-CO_2/O_2 battery. *ACS Catal.* **10**, 1640–1651 (2020).

14. Merum, D., Nallapureddy, R., R., Pallavolu, M. R., Mandal, T. K., Gutturu, R. R., Parvin, N., Banerjee, A. N., & Joo, S. W. Pseudocapacitive performance of freestanding $Ni_3V_2O_8$

nanosheets for high energy and power density asymmetric supercapacitors. *ACS Appl. Energy Mater.* **5**, 5561–5578 (2022).

15. Chen, G., Chen, D., Huang, J., Zhang, C., Chen, W., Li, T., Huang, B., Shao, T., Li, J., & Ostrikov, K. K. Focused plasma-and pure water-enabled, electrode-emerged nanointerfaced NiCo hydroxide–oxide for robust overall water splitting. *ACS Appl. Mater. Interfaces* **13,** 45566–45577 (2021).

16. Li, R., Rao, D., Zhou, J., Wu, G., Wang, G., Zhu, Z., Han, X., Sun, R., Li, H., Wang, C., Yan, W., Zheng, X., Cui, P., Wu, Y., Wang, G., & Hong, X. Amorphization-induced surface electronic states modulation of cobaltous oxide nanosheets for lithium-sulfur batteries. *Nat Commun* **12**, 3102 (2021).

17. Hatanaka, M., Takahashi, N., Takahashi, N., Tanabe, T., Nagai, Y., Suda, A., & Shinjoh. H. Reversible changes in the Pt oxidation state and nanostructure on a ceria-based supported Pt. *J. Catal.* **266**, 182–190 (2009).

18. Qin, J.H., Xu, P., Huang, Y.D., Xiao, L.Y., Lu, W., Yang, X.G., Ma, L.F., & Zang, S.Q. High loading of Mn(II)-metalated porphyrin in a MOF for photocatalytic CO_2 reduction in gas–solid conditions. *Chem. Commun.* **57**, 8468-8471 (2021).

19. Ding, Y., Chen, Y., Guan, Z., Zhao, Y., Lin, J., Jiao, Y., & Tian, G. Hierarchical CuS@$ZnIn_2S_4$ hollow double-shelled p–n heterojunction octahedra decorated with fullerene C60 for remarkable selectivity and activity of CO_2 photoreduction into CH_4. *ACS Appl. Mater. Interfaces* **14**, 7888–7899 (2022).

20. Bi, W., Hu, Y., Jiang, H., Zhang, L., & Li, C. Revealing the sudden alternation in Pt@h-BN nanoreactors for nearly 100% CO_2-to-CH_4 photoreduction. *Adv. Funct. Mater.* **31***,* 2010780 (2021).

21. She, H., Zhou, H., Li, L., Zhao, Z., Jiang, M., Huang, J., Wang, L., & Wang, Q. Construction of a two-dimensional composite derived from TiO_2 and SnS_2 for enhanced photocatalytic reduction of CO_2 into CH_4. *ACS Sustainable Chem. Eng.* **7**, 650−659 (2019).

22. Wang, Y., Zhang, Z., Zhang, L., Luo, Z., Shen, J., Lin, H., Long, J., Wu, J. C. S., Fu, X., Wang, X., & Li, C. Visible-Light Driven Overall Conversion of CO_2 and H_2O to CH_4 and O_2 on 3D-SiC@2D-MoS_2 Heterostructure. *J. Am. Chem. Soc.* **140**, 14595–14598 (2018).

23. Chen, K., Jiang, T., Liu, T., Yu, J., Zhou, S., Ali, A., Wang, S., Liu, Y., Zhu, L., & Xu, X. Zn dopants synergistic oxygen vacancy boosts ultrathin CoO layer for CO_2 photoreduction. *Adv. Funct. Mater.* **32***,* 2109336 (2022).

24. Chai, Y., Chen, Y., Shen, J., Ni, M., Wang, B., Li, D., Zhang, Z., & Wang, X. Distortion of the coordination structure and high symmetry of the crystal structure in In_4SnS_8 microflowers for enhancing visible-light photocatalytic CO_2 reduction. *ACS Catal.* **11**, 11029–11039 (2021).

25. Li, X., Sun, Y., Xu, J., Shao, Y., Wu, J., Xu, X., Pan, Y., Ju, H., Zhu, J., & Xie, Y. Selective visible-light-driven photocatalytic CO_2 reduction to CH_4 mediated by atomically thin $CuIn_5S_8$ layers. *Nat. Energy* **4**, 690–699 (2019).

26. Deng, Y., Wan, C., Li, C., Wang, Y., Mu, X., Liu, W., Huang, Y., Wong, P. K., & Ye, L. Synergy effect between facet and zero-valent copper for selectivity photocatalytic methane formation from CO_2. *ACS Catal.* **12**, 4526–4533 (2022).

27. Yin, G., Huang, X., Chen, T., Zhao, W., Bi, Q., Xu, J., Han, Y., & Huang, F. Hydrogenated blue titania for efficient solar to chemical conversions: preparation, characterization, and reaction mechanism of CO_2 reduction. *ACS Catal.* **8**, 1009–1017 (2018).

28. Tang, Z., He, W., Wang, Y., Wei, Y., Yu, X., Xiong, J., Wang, X., Zhang, X., Zhao, Z., & Liu, J. Ternary heterojunction in rGO-coated Ag/Cu_2O catalysts for boosting selective photocatalytic CO_2 reduction into CH_4. *Appl. Catal., B* **311**, 121371 (2022).

29. Wang, X.D., Huang, Y.H., Liao, J.F., Jiang, Y., Zhou, L., Zhang, X.Y. Chen, H.Y., & Kuang, D.-B. In situ construction of a Cs_2SnI_6 perovskite nanocrystal/SnS_2 nanosheet heterojunction with boosted interfacial charge transfer. *J. Am. Chem. Soc.* **141**, 13434–13441 (2019).

30. Xiong, Z., Lei, Z., Kuang, C. C., Chen, X., Gong, B., Zhao, Y., Zhang, J., Zheng, C., & Wu, J. C.S. Selective photocatalytic reduction of CO_2 into CH_4 over Pt-Cu_2O TiO_2 nanocrystals: The interaction between Pt and Cu_2O cocatalysts. *Appl. Catal., B* **202**, 695–703 (2017).

31. Bie, C., Zhu, B., Xu, F., Zhang, L., & Yu, J. In situ grown monolayer N-doped graphene on CdS hollow spheres with seamless contact for photocatalytic CO_2 reduction. *Adv. Mater.* **31***,* 1902868 (2019).

32. Cao, Y., Guo, L., Dan, M., Doronkin, D. E., Han, C., Rao, Z., Liu, Y., Meng, J., Huang, Z., Zheng, K., Chen, P., Dong, F., & Zhou, Y. Modulating electron density of vacancy site by single Au atom for effective CO_2 photoreduction. *Nat. Commun.* **12**, 1675 (2021).

33. Xu, J., Ju, Z., Zhang, W., Pan, Y., Zhu, J., Mao, J., Zheng, X., Fu, Ha., Yuan, M., Chen, H., & Li, R. Efficient infrared-light-driven CO_2 reduction over ultrathin metallic Ni-doped CoS_2 Nanosheets. *Angew. Chem. Int. Ed.* **60**, 8705–8709 (2021).

34. Long, R., Li, Y., Liu, Y., Chen, S., Zheng, X., Gao, C., He, C., Chen, N., Qi, Z., Song, L., Jiang, J., Zhu, J., & Xiong, Y. Isolation of Cu atoms in Pd lattice: forming highly selective sites for photocatalytic conversion of CO_2 to CH_4. *J. Am. Chem. Soc.* **139**, 4486–4492 (2017).

35. Tan, D., Zhang, J., Shi, J., Li, S., Zhang, B., Tan, X., Zhang, F., Liu, L., Shao, D., & Han, B. Photocatalytic CO_2 transformation to CH_4 by Ag/Pd bimetals supported on N-Doped TiO_2 nanosheet. *ACS Appl. Mater. Interfaces* **10**, 24516–24522 (2018).
